# Is the Greener Approach Better? Application of Electrochemistry in the Synthesis of Perylenediimides

**DOI:** 10.3390/molecules30132683

**Published:** 2025-06-21

**Authors:** Patrycja Filipek, Agata Szlapa-Kula, Stanisław Krompiec, Krzysztof Zemlak, Bartłomiej Kula, Karol Erfurt, Michał Filapek

**Affiliations:** 1 Institute of Chemistry, Faculty of Science and Technology, University of Silesia, Szkolna 9, 40-007 Katowice, Poland; agata.szlapa-kula@us.edu.pl (A.S.-K.); stanislaw.krompiec@us.edu.pl (S.K.); 2 Syntal Chemicals Sp. z o.o., ul Łabędzka 59, 44-121 Gliwice, Poland; 3 Department of Chemical Organic Technology and Petrochemistry, Silesian University of Technology, B. Krzywoustego 4, 44-100 Gliwice, Poland; karol.erfurt@polsl.pl

**Keywords:** perylenediimides, electrochemistry, synthesis, electrochromism

## Abstract

Perylenediimides are an interesting group of compounds that are finding more and more applications. However, the synthetic route of obtaining and modifying them is usually very complicated, costly, and time-consuming. Therefore, the conducted research aimed to develop new, greener, electrochemical methods of obtaining unknown perylenediimides (containing 2-ethylhexyl at the nitrogen atom). For the products obtained in this way, optical and electrochemical studies were conducted and compared with DFT results (i.e., energy gaps and HOMO and LUMO levels). Asa result of optical studies, different emission wavelengths of two isomers originating from the same excitation wavelength were observed. Electrochemical studies also confirmed significant differences in properties between the obtained isomers. Spectroelectrochemical measurements were also performed; they revealed the electrochromic properties of the obtained isomers in the visible and near-infrared range. Considering all the properties (optical and (spectro)electrochemical), the obtained compounds have a high potential for use in optoelectronic devices. Moreover, unprecedented pi-expansion of *cis*-DBPDI via 1,2-bis(p-bromophenyl)acetylene Diels–Alder cycloaddition into the bay region was also realized successfully. Summing up, electrosynthesis and further pi-expansion via cycloaddition offer a sea of opportunities for obtaining nanographenes.

## 1. Introduction

Perylenediimides (PDIs) are classical high-performance dyes invented and described in 1913. They are characterized by good chemical, thermal, and light stability and resistance to weathering [1]. Over the next hundred years, these compounds were widely studied. As a result, strong absorption of these compounds in the visible region was observed due to large pi couplings, high electron affinity resulting from electron-deficient imide groups, and good electron transport properties due to effective electron coupling between π systems on adjacent planar molecules. This last feature opened up new avenues for applying PDI, e.g., in solar cells [2]. Despite the presence of various photovoltaic technologies, such as quantum dot solar cells or perovskite solar cells [3,4,5], organic solar cells still represent the most advantages, such as lightness, flexibility, or the possibility of modulating appropriate energy levels through functionalization [6,7]. In addition to solar cells, PDIs are widely used in photovoltaic devices or field-effect transistors (OFETs) [8]. They are also increasingly used in bioimaging, biosensing, and photodynamic therapy [9,10].

However, despite their excellent properties, PDI synthesis is a long and often multi-stage process that requires consideration of many factors to obtain the best possible final product. To make PDIs and their extended derivatives DBPDIs (dibenzo perylene diimides) soluble, functionalization in the imide position with alkyl groups is required [9,10,11]. At a later stage, dimerization proved to be the most effective method to obtain PDI. In 2008, Langhals and his co-workers carried out a series of syntheses to obtain anthracene-1,9-dicarboximide. The starting structure was anthracene, from which anthracene carboximide (which has excellent optical properties) was obtained after a series of transformations. The intermediate was then dimerized using molten KOH. As a result, anthracene gel was obtained [12]. Yao and his group also attempted to synthesize this compound a year later. The synthetic route was practically the same, and the basic (starting) structure was also the same. The final products of these two groups differed in the substituents at the nitrogen atom [13]. As can already be seen, developing a new synthetic method for obtaining PDI derivatives is extremely important. In the field of synthesis, one of the few methods that allow the elimination of solid bases such as KOH or t-BuOK from the synthesis of DBPDI is electrochemistry.

The first industrial use of electrolysis dates back to the end of the 19th century. Despite this, few electrochemistry cases have been applied to organic synthesis. This fact is surprising because this technique is characterized by relatively mild conditions, good tolerance of functional groups, and high chemoselectivity. In addition, the ease with which many electrochemical reactions can be scaled up and the intrinsic “greenness” of the responses (since an electric current is used instead of stoichiometric oxidants or reductants) make this chemistry attractive in the context of process chemistry. These advantages, however, do not overcome the concerns of organic chemists who fear electrochemistry due to the perceived complexity of the reaction configuration (potentiostat, divided/undivided cells, electrode composition, type of experiment, etc.) and the seemingly infinite number of reaction variables (electrolyte, electrode composition, cell type, etc.) [14,15]. However, this approach has yet to be abandoned.

In 2017, J. Jiang and his group decided to use electrochemistry to synthesize heterocycles. Heterocycles are one of the largest groups of organic compounds with a wide range of applications in pharmaceuticals, organic materials, and biologically active materials [16]. The synthesis of such structures often requires the use of expensive reagents, harsh conditions, and long reaction times, and generates large amounts of waste. Therefore, electrochemical techniques are a great alternative [17]. Electrochemical reactions are driven by single-electron transfer processes initiated at the electrode surface. Hence, electrochemical transformations can be considered heterogeneous reactions, and substrates or electron mediators must be transported from the bulk solution to the electrode surface. Typically, an electrochemical reaction starts with the mass transfer of the substrate from the bulk phase to the heterogeneous electrode surface. Then, the substrate adsorbs on the electrode, and electron transfer can occur, converting the substrate into a product. Finally, the product is desorbed from the electrode and diffuses back to the liquid phase [18].

In addition to synthesizing heterocyclic compounds containing only carbon skeletons, it is possible for electrosynthesis to obtain N-, S-, and O-doped heterocyclic compounds. In 2021, He and his group synthesized iodinated and brominated N-heterocycles by electrochemical oxidation. This undertaking was successful. The process was carried out in an indivisible cell, where platinum played the role of the cathode and graphite the anode. The products of this reaction could be subject to further functionalization [19]. In 2019, Dong and his group conducted electrochemical thiolation of aryl bromides and chlorides catalyzed by nickel. The process was carried out at room temperature. The role of the cathode was played by a nickel electrode and the role of the anode by a magnesium electrode. The process was carried out with good efficiency, up to 95% [20,21]. In 2019, Guan and his group carried out oxidative cyclization of olefins, resulting in an oxygen atom in the ring. He also carried out several other reactions of this type. Each was successful, and their efficiency was around 80–90%. These types of reactions are described in detail in the review by Martins et al. [22,23].

Electrochromic materials have flourished in recent decades. Electrochromism refers to the property of some materials that undergo an electrochemical color change, i.e., a reversible optical change (absorbance, transmission, and reflection) triggered by electron transfer processes of some materials under the influence of small electric fields [24]. The term itself was developed and introduced in 1961 by J. R. Platt [25]. Due to such properties, modules based on electrochromism are widely used in various devices such as smart windows or temperature-controlled buildings [26,27].

Therefore, the natural next step was to check whether PDI, due to its excellent electronic conductivity, would also exhibit electrochromic properties. Tao and his group developed two derivatives, one based on PDI. The layer based on this derivative (ZnO@PDI-COOH) gave the best result at a low voltage, allowing reversible color switching (from red to violet) from −0.6 to 0 V. Moreover, the layer has excellent stability (retains 92.41% contrast after 2400 cycles) and afast switching time (dyeing time 1.7 s and bleaching time 2.6 s) [28].

**Scheme 1:**
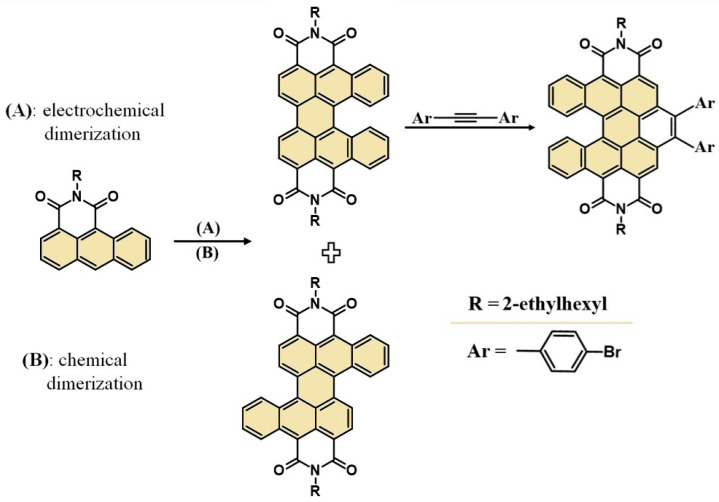
Synthesis of *cis*- and *trans*-DBPDI: electrochemical and chemical together with pi-expansion of *cis*-DBPDI.

Considering the current requirements for organic synthesis, we developed an unprecedented and eco-friendly electrochemical method for *cis*- and *trans*-DBPDI preparation—Scheme 1. Moreover, the derivatives obtained in this way can be successfully subjected to further pi-expansion, e.g., *cis*-DBPDI in a cycloaddition reaction to the bay region.

## 2. Results

**Figure 1:**
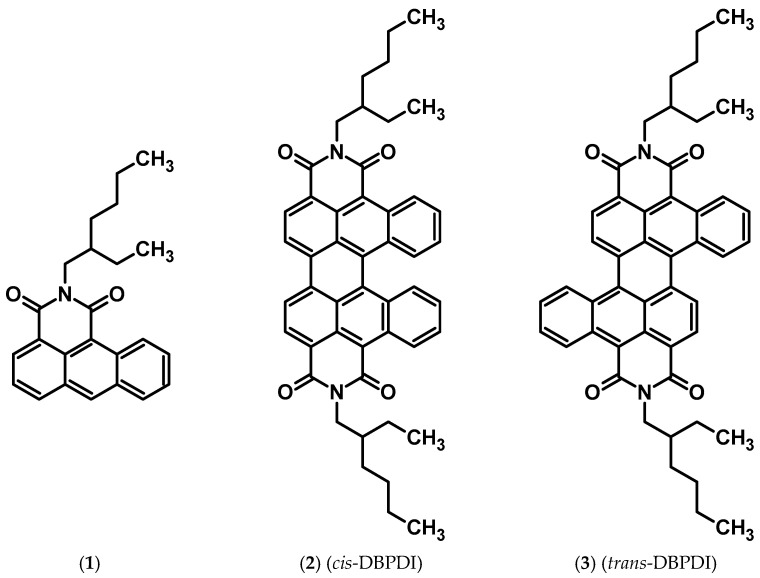
Structures of obtained compounds.

The main goal of this research was to present a new, alternative method of dibenzobisimide synthesis, which does not require the use of many harmful reagents. Structures of the obtained and evaluated compounds (whose NMR spectra are shown in the SI) are presented in Figure 1 (below).

**Scheme 2:**
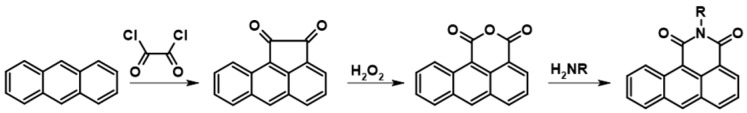
Schematic representation of the preparation of compound (**1**), where R = 2-ethylhexyl.

This work aimed to obtain fully soluble bisimides. Therefore, we decided to introduce a solubilizing 2-ethylhexyl moiety at the nitrogen atom (Scheme 2).

**Scheme 3:**
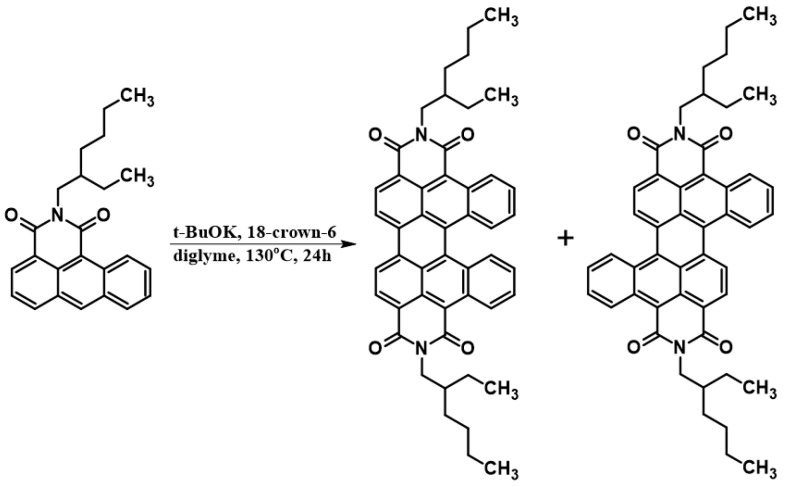
Schematic representation of the “classical” chemical synthesis reaction.

In the next step, compound **1** becomes a substrate subjected to a dimerization reaction. The pure chemical synthesis course (i.e.,without electrochemical methods) involves the necessity of using reagents such as t-BuOK and 18-crown-6 and heating (Scheme 3).

In contrast, the electrochemical method proposed in this paper does not require heating or using reagents other than the substrate, solvent, and electrolyte. In addition, a great advantage of the electrochemical process is that it takes place even in an air atmosphere. As a result of both methods, a mixture of cis and trans isomers is obtained. A schematic course of the electrochemical reaction mechanism is presented in SI (Scheme S1).

**Scheme 4:**
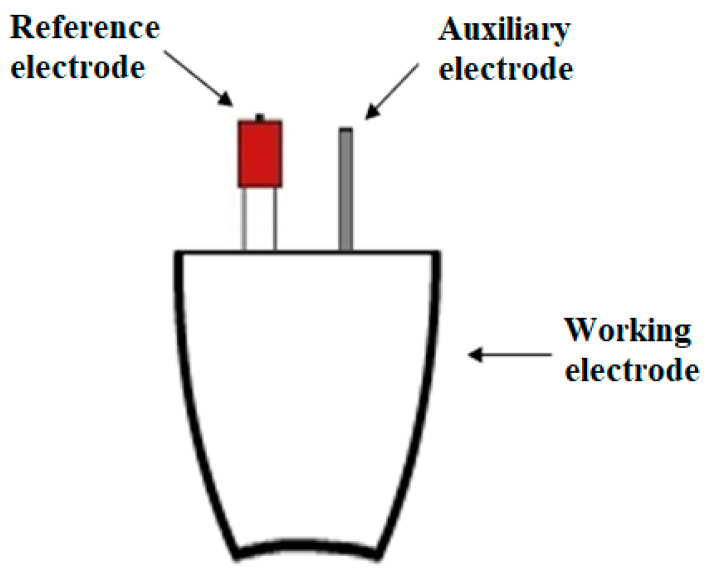
Schematic representation of the reactor in which the reaction takes place.

The entire electrochemical process occurs in a platinum crucible, which also acts as a reactor. The whole surface of the crucible acts as a working electrode, so the reaction takes place on the entire available surface. In addition to the working electrode, two other electrodes are placed in the solution: a reference electrode (made of silver) and an auxiliary electrode (platinum wire)—Scheme 4.

To ensure high conductivity, the process was carried out in a 0.1 M NBu_4_PF_6_ solution in CH_2_Cl_2_. During these studies, two methods of electrosynthesis were used: first, a 10 mA current was applied; second, a constant potential of 2 V was used. However, electrode polarization was forced in both cases, and an electron was detached from the substrate molecule, creating a radical cation. A specific type of transition state is then created (CIP1 or TIP1 in Scheme S1). Then, a single intermolecular bond is formed (with the elimination of the hydrogen). After the electrode is re-polarized, the above process is repeated (yielding CIP2 and TIP2 creation), resulting in a second bond formation, giving the final product as a mixture of two isomers. The entire process, both at constant voltage and current, is constantly monitored by TLC, whichis an additional advantage of the electrochemical method. Thus, this method is an example of the Scholl reaction using the principles of green chemistry. It meets the expectations of the principle of atom economy and the principle of limiting additional reagents. As mentioned earlier, in the case of classical dimerization methods, strong oxidants or a nitrogen base/tert-butanolate system are necessary. In the case of using the proposed synthesis method, the external potential forces the oxidation of the substrate molecule, initiating the reaction. This makes the reaction cleaner andcheaper and does not require the use of aggressive reagents. It is also worth emphasizing that the electrochemical method for obtaining perylenediimide has not been described to date.

### 2.1. DFT Calculation

To complement the experimental studies, theoretical studies were performed (presented in Table 1). Theoretical data were obtainedusing the Gaussian 16 program [29]. Quantum chemical calculations for the presented compounds were performed using the hybrid functional sets B3LYP [30,31] and 6-311 + G [32,33,34,35,36]. Structure optimization was performed in dichloromethane. TD-DFT calculations were performed based on these geometries to obtain UV–Vis absorption spectra. The experimental solvent solution was modeled using the polarizable continuum model [37,38].

In the first step, the frontier orbitals were analyzed. Table 1 shows the electronic probability density of the highest occupied molecular orbital (HOMO) and the lowest unoccupied molecular orbital (LUMO) calculated using the DFT approach. In the case of compound (**1**), the electron clouds of both orbitals encompass 1,9-anthracenedicarboxylic imide. For molecules (**2**) and (**3**), the modeling shows that both the HOMO and LUMO hybrid orbitals are mainly condensed on the core of the molecules (dibenzoperylenediimide fragment), with limited delocalization of the electron cloud onto the aliphatic chains. The ionization potential (IP) and electron affinity (EA) values were also calculated to compare the experimental results with theoretical values. The highest absolute IP value is characterized by the substrate (IP = 6.10 eV). Interestingly, *cis*-DBPDI (**2**) and *trans*-DBPDI (**3**) show practically the same IP energy values (Table 2). This shows that a small modification of the conformation does not have a large effect on the obtained results. These values are complementary to the experimentally obtained data, where IP for (**2**) = 5.89 eV and (**3**) = 5.77 eV. Both geometric isomers show improved results compared to the starting substrate (Table 2). In the case of EA, the value obtained from calculations for the *cis*-DBPDI molecule (**2**) and *trans*-DBPDI (**3**) increased significantly compared to compound (**1**). EA is −3.20 eV for (**1**), while for (**2**) and (**3**) it is −4.10 eV and −4.09 eV, respectively. Once again, one can observe a similarity in the values between derivatives (**2**) and (**3**). The experimental EA results show a similar trend to the theoretical calculations. According to TD-DFT calculations (TD-CAM-B3LYP/6-311 + G level, see Supporting Information Figures S2 and S3), the intense absorption band in the range of 600–800 nm for di-benzo perylenediimides can be assigned to the transition containing the main contribution of HOMO to LUMO (oscillator strength, f = 0.72 for (**2**) and f = 0.76 for (**3**)). In the case of (**1**) (f = 0.26), the band in the range of 400–500 nm also refers to the HOMO → LUMO transition. The reduced oscillator strength of (**1**) can be explained by the less effective overlap of the relatively localized HOMO and the well-delocalized LUMO. The consideration of the theoretical transition in the absorption band confirms the correct choice of the calculation conditions.

### 2.2. Photophysical and Thermal Properties

**Figure 2:**
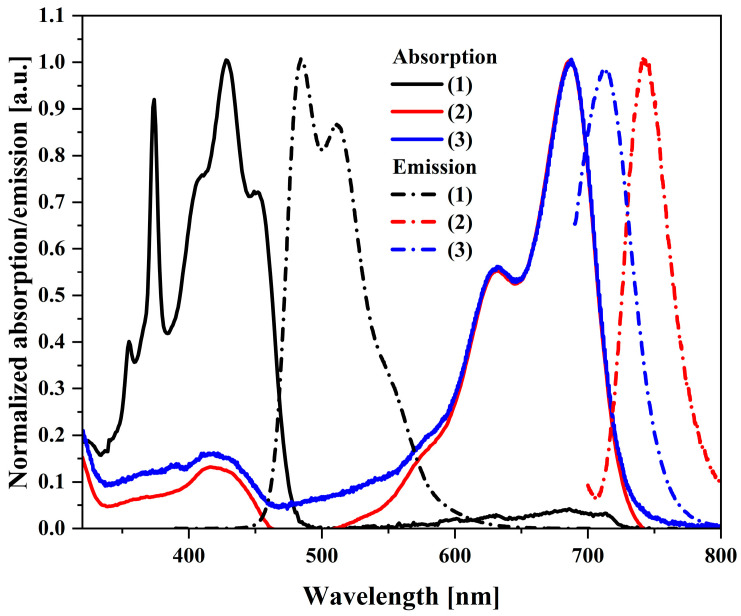
Normalized electronic absorption in CH_2_Cl_2_ (solid lines) and PL spectra of the investigated compounds (dotted lines); c = 2.5 × 10^−5^ mol/L.

The next step of the research was to perform UV–Vis and PL spectroscopy measurements in a diluted (2.5 × 10^−5^) solution in dichloromethane (Figure 2). Analyzing the obtained UV–Vis spectra, it can be seen that the maximum peak for the starting compound is between 350 and 450 nm. In comparison, the maximum absorbance of (**2**) is significantly shifted towards red waves and has a maximum at 687 nm, the same as the third compound (**3**). Both the starting compound and both isomers have small Stokes shifts (shown in Table S1). The smallest, 26 nm, is for the *trans*-DBPDI isomer. By analyzing the PL spectra, it is possible to observe different behavior of the compounds. Namely, the (**2**) and (**3**) isomers have one emission peak maximum at 744 nm and 714 nm, respectively. The shift towards near-infrared wavelengths is characteristic of bisimides [39]. Both bands are narrow, which may indicate a locally excited state. The exception is the emission band for the precursor, where there are two maxima (486 nm and 512 nm) with a quantum efficiency of 46%.

**Figure 3:**
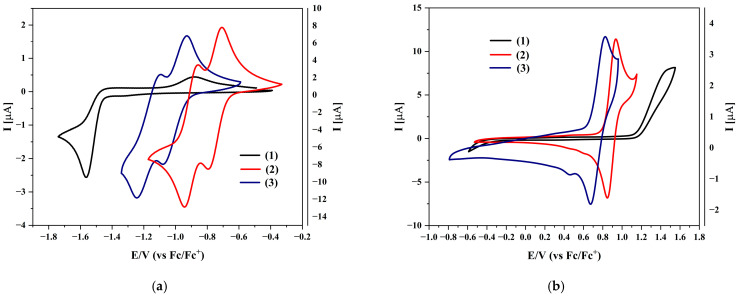
Cyclic voltammograms of the investigated compounds with sweep rate ν = 100 mV/s, 0.1 M Bu_4_NPF_6_ in CH_2_Cl_2_: (**a**) during negative potential sweeping; (**b**) during positive potential sweeping.

The optical properties were compared with the values derived from electrochemical measurements and DFT calculations (Table 2). As shown in Figure 3, the negative and positive sweep potential values are smaller for both isomers than for the starting compound. Reduction occurs most easily for compound (**2**), while oxidation occurs earlier for compound (**3**). It is also worth emphasizing that for compounds (**1**) and (**3**), the gap from electrochemical measurements is very close to the optical gap (for compound (**1**), it is practically the same). Moreover, both the reduction and oxidation processes are reversible, which opens up broader application possibilities for this type of compound.

The TGA curve (thermograms are presented in Figure S4 in the SI) showed that the dimerization product exhibited high thermal stability. The temperature of 5% mass loss (defined as one of the basic parameters characterizing the onset of thermal decomposition) was 386 °C, while that of the starting substrate was 244 °C.

### 2.3. Spectroelectrochemistry

The electrochemical measurements showed that compounds (**2**) and (**3**) are characterized by high stability of reduced and oxidized forms. To deepen the knowledge about these processes, a series of spectroelectrochemical measurements were performed (for comparison purposes, compound (**1**) was also tested). In the case of reduction of (**1**), the UV–Vis spectrum changes are insignificant (Figure S5). Only a slight shift towards the lower energy of the band can be observed. The maximum of this band after reduction shifts from 429 nm to 447 nm. In addition, two peaks disappear (with maxima at 353 and 373 nm), and instead of them, a new peak appears at 346 nm with a very high molar absorption coefficient. Therefore, reduction affects all electronic transitions (in the studied range) in this case.

**Figure 4:**
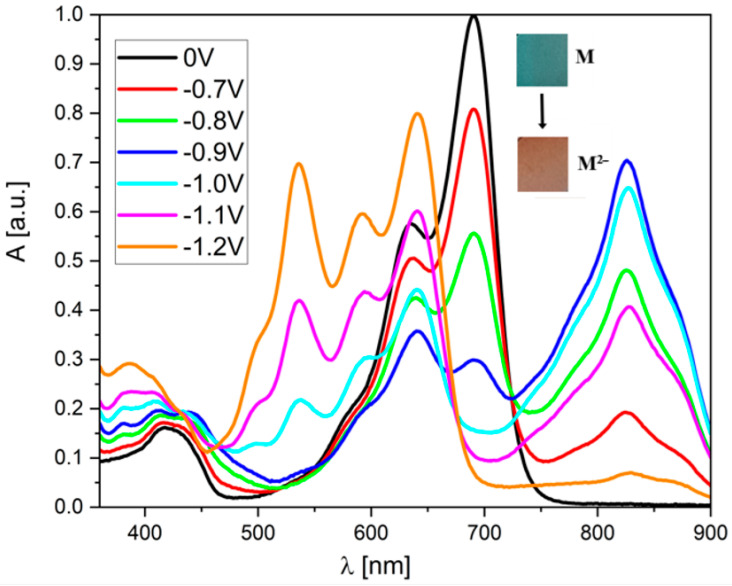
UV–Vis spectroelectrochemistry of the (**2**) derivative in CH_2_Cl_2_ solution during reduction (c = 1 × 10^−5^ mol/L, as an inset on each graph, all potentials vs. Fc/Fc^+^ redox coupe).

Much more significant changes occur when reducing derivatives (**2**) and (**3**). First of all, it must be noted that the molecules are more extensive from achemical point of view. Moreover, as electrochemical studies have shown, both of these derivatives have a “two-step” reduction, i.e., the reduction of the neutral N to the anion M−, and then the anion reduction of M− to the dianionic form M^2−^. When the potential reaches a value below E1red, visible changes in the UV–Vis spectrum of (**2**) can be observed (Figure 4). The band’s intensity, lying between 550 and 750, begins to decrease, while an additional peak appears with a maximum at 828 nm (for both (**2**) and (**3**)). The changes in the spectrum are the result of the electrochromism of the compound being tested. In its neutral form, it has a dark green color, but when reduced, it changes to red (see photos as an inset in Figure 4).

**Figure 5:**
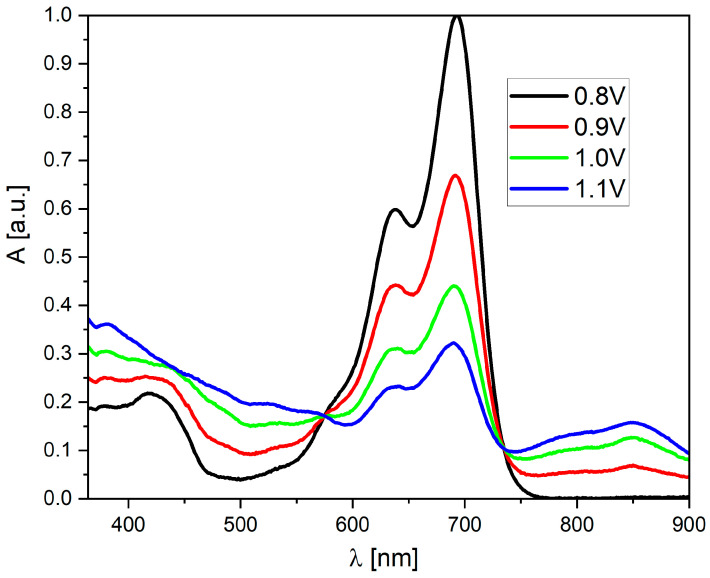
UV–Vis spectroelectrochemistry of the (**2**) derivative in CH_2_Cl_2_ solution during oxidation (c = 1 × 10^−5^ mol/L, as an inset on each graph, all potentials vs. Fc/Fc^+^ redox coupe).

Surprisingly, both molecules show significant differences in their properties after exceeding the E2red potential. For derivative (**3**), one can observe a further increase in the band’s intensity at 828 nm (Figure S6), which becomes dominant in the entire spectrum (highest molar coefficient). The intensity of the broadband from 380 to 600 nm also increases, and two additional local maxima appear at 537 and 592 nm. Additionally, the peak at 642 nm is also “recreated.” Interestingly, the peak with a maximum at 688 nm (present in the spectrum of the neutral compound) disappears completely. In contrast, in the case of derivative (**2**), after exceeding the E2red potential, the band with a maximum at 824 nm disappears completely (which is the most significant difference compared to the “trans” derivative). In this case, a very intense band is formed in the 450–700 nm range with three clearly visible maxima at 535, 590, and 639 nm. However, the common feature of (**2**) and (**3**) is the fact that the most intense peak for their neutral form (with λ(max) = 690 nm) disappears. On the other hand, the behavior of both derivatives during oxidation is very similar—a broadband is formed without a distinct maximum, which is characteristic of oxidized forms in molecules with a large number of conjugated double bonds (Figure 5).

### 2.4. Further Functionalisation via Diels–Alder Cycloaddition into Bay Region—Preliminary Tests

**Scheme 5:**
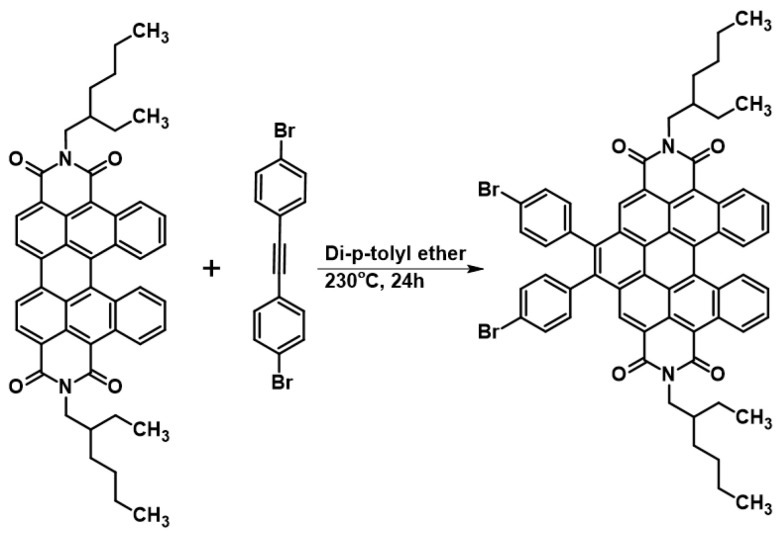
Pi-expansion of *cis*-DBPDI via 1,2-bis(p-bromophenyl)acetylene DAC into bay region (**4**).

Finally, preliminary studies were carried out on the possibilities of further expansion of the aromatic fragment of the obtained derivatives. It is well known that PDI, and other PAH pi-expansion via DAC, is an especially attractive strategy applied for various types of modifications of the above-mentioned structures. However, DAC into the *cis*-DBPDI bay region is totally unknown. During the preliminary tests on further π-expansion, we managed to successfully perform a reaction involving *cis*-DBPDI and 1,2-bis(p-bromophenyl)acetylene, see Scheme 5. Dienophile was selected, taking into consideration our experiences in the field ofDAC into the PAH bay region [40]. Using an acetylene derivative containing bromine makes even further functionalization of the molecule possible, e.g., by coupling reactions or cycloaromatization. This confirms that the synthetic strategy presented in this work not only allows obtaining compounds that are interesting in themselves, but is also a bridge between simple anthracene derivatives and functionalized nanographenes.

## 3. Materials and Methods

### 3.1. Reagents and Materials

**General Methods:** All chemicals and starting materials were commercially available and were used without further purification. Solvents were distilled as per the standard methods and purged with nitrogen before use. All reactions and measurements were carried out under an argon atmosphere unless otherwise indicated. Column chromatography was carried out on Merck Silica Gel 60 (Darmstadt, Germany). Thin-layer chromatography (TLC) was performed on silica gel (Merck TLC Silica Gel 60 F254). ^1^H NMR spectra were recorded using a Bruker AvanceUltraShield 400 MHz spectrometer (Billerica, MA, USA). The peaks were referenced to the residual CDCl3 (7.28 ppm) resonances in ^1^H and ^13^C NMR spectra, respectively. UV/Vis spectra were recorded with a Hewlett–Packard model 8453 UV/Vis spectrophotometer (Palo Alto, CA, USA) in dichloromethane solution. Electrochemical measurements were carried out with an Eco ChemieAutolab PGSTAT128n potentiostat (Herisau, Switzerland) using glassy carbon (with diam. 2 mm) as the working electrode, while platinum coil and silver wire were used as the auxiliary and reference electrodes, respectively. Potentials are referenced with respect to ferrocene (Fc), which was used as the internal standard. Cyclic and differential pulse voltammetry experiments were conducted in a standard one-compartment cell in CH_2_Cl_2_ (Carlo Erba, HPLC grade, Milan, Italy) under argon. Bu4NPF6 (Aldrich; 0.2 M, 99%, St. Louis, MO, USA) was used as the supporting electrolyte. Waters Xevo G2 Q-TOF (Waters Corporation, Milford, MA, USA) equipped with an ESI source operated in positive ion mode. Full-scan MS data were collected from 100 to 5000 Da in positive ion mode with a scan time of 0.1 s. To ensure accurate mass measurements, data were collected in centroid mode, and mass was corrected during acquisition using a leucine enkephalin solution as an external reference (Lock-Spray^TM^, Chattanooga, TN, USA), which generated a reference ion at *m*/*z* 556.2771 Da ([M + H]^+^) in positive ESI mode. Accurate mass and molecular ion adduct composition were calculated using MassLynx 4.2 software (Waters) supplied with the instrument. Quantum theoretical calculations were performed with the use of density functional theory (DFT), with exchange–correlation hybrid functionals B3LYP and CAM B3LYP; the basis 6-311 + G was used for all atoms. The calculations were carried out with the use of the Gaussian 16 program.

### 3.2. Synthesis of N-(2-Ethylhexyl)anthraceneimide (***1***)

2-Ethylhexylamine (27.93 g), dimethylformamide (218 mL), and anthracene-1,2-dicarboxylic acid anhydride (10.72 g) were placed in a glass reactor (500 mL) equipped with a reflux condenser and a magnetic stirrer under Ar. The resulting reaction mixture was stirred at 135 °C for 18 h. After cooling to room temperature, the reaction mixture was poured into a mixture of 1 M HCl (1600 mL) with CH_2_Cl_2_ (800 mL). The resulting biphasic mixture was vigorously stirred for 15 min, and then the organic layer was separated. The aqueous layer was extracted with CH_2_Cl_2_ (500 mL). Both extracts were combined and then washed with 1 M HCl (600 mL) and finally with H_2_O (800 mL). After drying with anhydrous MgSO_4_, the volatile part of the dry extract was evaporated using a vacuum rotary evaporator. The obtained dark oil (44 g) was purified by chromatography (column length 61 cm, diameter 4.5 cm, silica gel 40–63 µm ca. 700 mL, eluent CH_2_Cl_2_). Finally, 11.93 g (73% yield) of N-(2-ethylhexyl)anthracene-1,2-dicarboxylic acid imide was obtained as a brown powder. Yield: 73%; HRMS EI MS: [M+] calcd for C_24_H_25_NO_2_: 359.1885, found: 359.3158.

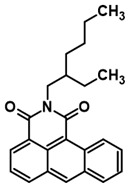



**^1^H NMR** (400 MHz, CDCl_3_) δ 9.73 (d. *J* = 9.1 Hz. 1H), 8.49 (d. *J* = 6.8 Hz. 1H), 8.36 (s.1H), 7.99 (d. *J* = 8.3 Hz. 1H), 7.78 (d. *J* = 8.3 Hz. 1H), 7.64–7.59 (m. 1H), 7.51–7.45 (m. 1H), 7.45–7.39 (m. 1H), 5.25 (s. 1H), 4.09 (qd. *J* = 12.8. 7.4 Hz. 2H), 1.96 (dd. *J* = 12.5. 5.9 Hz. 1H), 1.48–1.37 (m, 4H), 1.37–1.24 (m, 4H),0.94 (t. *J* = 7.4 Hz. 3H), 0.87 (t. *J* = 7.0 Hz. 3H). **^13^C NMR** (126 Hz, CDCl_3_) δ 165.29, 163.96, 135.89, 134.71, 133.30, 132.25, 131.08, 129.57, 128.59, 126.80, 126.29, 125.29, 122.43, 115.23, 44.20, 37.96, 30.82, 28.73, 24.11, 23.17, 14.15, 10.74.

### 3.3. Synthesis of N,N’-bis(2-Ethylhexyl)-cis-dibenzoperylene Diimide (***2***) and N,N’-bis(2-Ethylhexyl)-trans-dibenzoperylene Diimide (***3***)

The synthetic procedure was realized under argon atmosphere: 1 mmol substrate, i.e., N-(2-ethylhexyl)-anthracenecarboxyimide, and 10 mL of solvent, i.e., ethylene glycol dimethyl ether, were placed in a glass reactor equipped with a magnetic stirrer. Next, 4,5 mmol 18-crown-6 was added and the mixture was heated at 130 °C for 15 min. After 15 min, 6 mmol potassium tert-butanolatewasadded to the stirred reaction mixture. The reaction was stirredfor 24 h. The post-reaction mixture was cooled down to room temperature and dissolved in DCM (50 mL). Next, the obtained solution was washed twice with HCl (2 × 5 mL, 35%) and also twice with water (2 × 150 mL). After drying with anhydrous MgSO_4_, the solution underwent typical chromatography on silica gel (150 mL). The desired product, namely the mixture of N,N’-bis(2-ethylhexyl)-*cis*-dibenzoperylenediimide and N,N’-bis(2-ethylhexyl)-*trans*-dibenzoperylenediimide, was obtained in the last fraction. After the second chromatography, pure N,N’-bis(2-ethylhexyl)-*cis*-dibenzoperylenediimide and pure N,N’-bis(2-ethylhexyl)-*trans*-dibenzoperylenediimide were obtained (DCM as an eluent) as separate fractions. Yield: 18%; HRMS EI MS: [M+] calcd for C_48_H_46_N_2_O_4_: 714.3458, found: 714.3466.

### 3.4. GalvanostaticSynthesis of N,N’-bis(2-Ethylhexyl)-cis-dibenzoperylene Diimide (***2***) and N,N’-bis(2-Ethylhexyl)-trans-dibenzoperylene Diimide (***3***)

The procedure was carried out in an air atmosphere at a temperature of 20–25 °C: 1 mmol of N-(2-ethylhexyl)anthraceneimide and 20 mL of CH_2_Cl_2_ were placed in a platinum crucible (acting as a reactor), equipped with a magnetic stirrer, with reference and auxiliary electrodes (acting as a cathode) both made of Pt wire. Then, 0.05 mmol of potassium tert-butoxide and 1.75 mmol of electrolyte (Bu4NPF6) were added to the constantly stirred reaction mixture. Then, the electrochemical process was carried out, controlled by a galvanostat, using the following parameters: 10 mA for 5000 s. After switching off the current, the substrate and product oxidized forms were transformed into neutral forms. The mentioned process lasted for 400 s and was repeated four times. Finally, the desired product was obtained by separating the reaction mixture using column chromatography on silica gel and CH_2_Cl_2_ with hexane (1/3: *v*/*v*) as the eluent.

### 3.5. Potentiostatic Synthesis of N,N’-bis(2-Ethylhexyl)-cis-dibenzoperylene Diimide (***2***) and N,N’-bis(2-Ethylhexyl)-trans-dibenzoperylene Diimide (***3***)

The procedure was conducted in an air atmosphere at a temperature of 20–25 °C. An amount of 1 mmol of N-(2-ethylhexyl)anthraceneimide and 60 mL of CH_2_Cl_2_ were placed in a platinum crucible (reactor-electrolyte) equipped with a magnetic stirrer, with reference and auxiliary electrodes (made of Pt wire). In the next step, 0.05 mmol of potassium tert-butoxide and 4.6 mmol of electrolyte (Bu_4_NPF_6_) were added to the constantly stirred reaction mixture. After establishing the oxidation peak at 1.9 V in the initial measurement (using differential pulse voltammetry), the electrochemical process was carried out using an anodic potential of 2 V. After 10 s, a reduction potential of −0.5 V was applied for another 20 s. The above-defined/described oxidation–reduction cycle was repeated four times. Finally, the reaction mixture was separated using silica gel column chromatography and CH_2_Cl_2_ with hexane (1/3: *v*/*v*) to afford the desired product. Yield: 16–18% (depends on procedure); HRMS EI MS: [M+] calcd for C_48_H_46_N_2_O_4_: 714.3458, found: 714.3466.

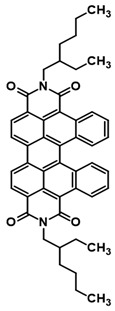



**^1^H NMR** (400 MHz, CDCl_3_) δ 9.86 (d. *J* = 9.0 Hz. 2H), 8.83 (d. *J* = 7.7 Hz. 2H), 8.59 (d. *J* = 7.8 Hz. 2H), 7.82–7.70 (m. 2H), 7.53 (d. *J* = 8.7 Hz. 2H), 7.32 (d. *J* = 7.4 Hz. 2H), 4.42–4.22 (m. 4H), 2.16–2.04 (m. 2H), 1.56–1.42 (m. 8H), 1.42–1.30 (m, 8H), 1.02 (t. *J* = 7.4 Hz. 6H), 0.93 (t. *J* = 7.0 Hz. 6H). **^13^C NMR** (126 MHz, CDCl3) δ: 164.54, 163.44, 133.89, 133.49, 133.22, 132.03, 131.45, 130.71, 128.56, 127.22, 127.08, 126.87, 124.76, 122.73, 122.46, 117.12, 44.53, 38.10, 30.87, 29.70, 28.73, 23.17, 14.17, 10.75.

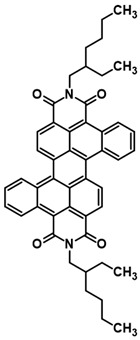



**^1^H NMR** (500 MHz, CDCl_3_) δ 10.07 (d. *J* = 9.0 Hz. 2H), 8.83 (d. *J* = 7.8 Hz. 2H), 8.68 (d. *J* = 8.6 Hz. 2H), 8.49 (d. *J* = 7.8 Hz. 2H), 7.93–7.89 (m. 2H), 7.79–7.75 (m. 2H), 4.32 (qd. *J* = 12.9. 7.4 Hz. 4H), 2.14–2.05 (m, 2H), 1.53–1.46 (m, 8H), 1.34–1.27 (m, 8H), 1.04–0.98 (t, 6H), 0.95–0.85 (t, 6H). **^13^C NMR** (126 MHz, CDCl_3_) δ 164.77, 163.46, 134.31, 133.82, 133.55, 131.53, 130.92, 130.58, 128.98, 128.24, 127.76, 126.28, 124.48, 122.27, 67.99, 53.43, 44.56, 38.09, 34.68, 34.14, 31.60, 30.88, 28.76, 26.92, 25.62, 24.11, 23.18, 22.67, 22.35, 14.07, 10.76.

### 3.6. Synthesis of 1-2;5-6-Dibenzo-8,11-cis[1′,2′-bis(p-bromophenyl)ethen-1′,2′-diyl]-N,N’-(2-ethylohexyl)perylenediimide (***4***)

An amount of 1.00 mmol of N,N’-bis(2-ethylhexyl)-*cis*-dibenzoperylenediimide, 4.00 mmol of 1,2-bis(4-bromophenyl)acetylene, and 10.0 mmol of flux, i.e., di-p-tolyl ether, were introduced into a glass ampoule resistant to overpressure up to 2 atmospheres. After creating a vacuum in the ampoule below 0.01 Pa and submerging the ampoule, the reaction mixture was subjected to homogenization. For this purpose, it was heated in an oil bath, from ambient temperature up to 160 °C, at a rate of 5–10 °C/min, occasionally mixing the contents of the ampoule by shaking, and then held at the final temperature (i.e., at 160 °C) until homogeneity was achieved, which occurred in the total time of 20 min. Then, the reaction mixture was heated in the ampoule at 230 °C for 24 h, without stirring. After cooling the post-reaction mixture to a temperature of approximately 20 °C, it was dissolved in 12 mL of CH_2_Cl_2_ and subjected to column chromatography on silica gel. Elution was carried out using a mixture of CH_2_Cl_2_ and hexane (1/5: *v*/*v*). Yield: 24%; HRMS EI MS: [M+] calcd for C_62_H_54_Br_2_N_2_O_4_: 1046.2294, found: 1047.1428.

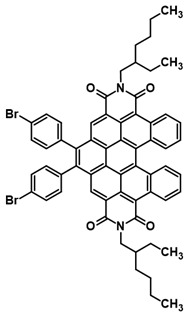



**^1^H NMR** (400 MHz, CDCl_3_) δ 10.03 (d, *J* = 8.9 Hz, 2H), 9.14 (s, 2H), 8.31 (d, *J* = 8.6 Hz, 2H), 7.90 (td, *J* = 7.0, 3.3 Hz, 2H), 7.58–7.47 (m, 6H), 7.17 (d, *J* = 8.4 Hz, 4H), 4.34–4.12 (m, 4H), 2.11–1.99 (m, 2H), 1.49–1.42 (m, 8H), 1.41–1.21 (m, 8H), 0.96 (t, *J* = 7.3, 4.3 Hz, 6H), 0.87 (t, *J* = 6.6 Hz, 6H). **^13^C NMR** (101 MHz, cdcl3) δ 164.92, 163.79, 140.87, 136.08, 133.83, 132.87, 132.73, 131.64, 131.59, 131.22, 131.02, 130.01, 129.92, 129.25, 128.63, 126.84, 126.78, 126.40, 123.53, 123.06, 122.88, 122.63, 117.54, 44.72, 38.06, 38.03, 30.88, 30.84, 29.66, 28.78, 28.69, 26.88, 24.17, 24.15, 23.15, 23.09, 14.07, 10.74, 10.69.

## 4. Conclusions

In the presented studies, two isomeric dimers of anthracene imide were obtained using electrochemical synthesis. DFT measurements were performed to determine the energy parameters (such as IP, Ea, and energy gap). During the physicochemical measurements, it was observed that the obtained isomers differ significantly from the starting compound and from each other. As a result of optical studies, different emission wavelengths of the two isomers originating from the same excitation wavelength were observed. Electrochemical studies showed that the *trans*-DBPDI isomer undergoes oxidation more easily, while the *cis*-DBPDI isomer undergoes reduction more easily. Spectroelectrochemical measurements were also performed; they confirmed the electrochromic properties of the obtained isomers in the visible and near-infrared range. Considering all the tested properties (optical and (spectro)electrochemical), the obtained compounds have a high potential for use in electrochromic devices. Moreover, further pi-expansion of *cis*-DBPDI via Diels–Alder cycloaddition opens anew area in the chemistry of dibenzoperylenediimides.

## Figures and Tables

**Table 1:** The HOMO and LUMO levels.

Code	HOMO	LUMO
(**1**)	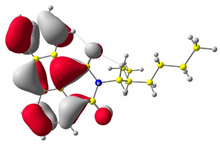	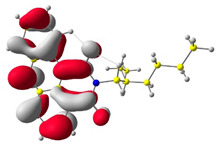
(**2**)	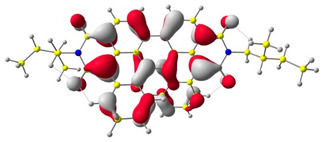	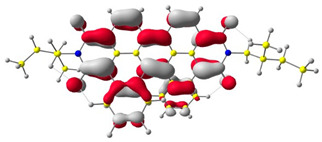
(**3**)	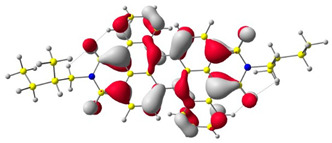	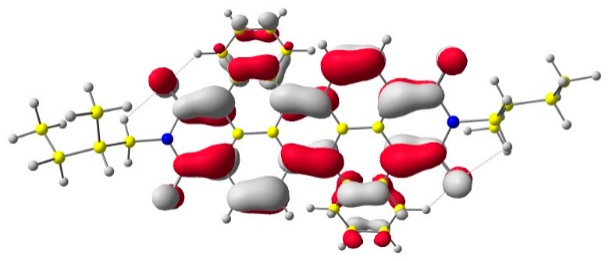

**Table 2:** IP, EA, and energy gap values for the compounds (**1**)–(**3**).

Code	Eox	Ered	IP ^a^	EA ^a^	Eg (CV) ^a^	Eg (Opt) ^b^	IP ^c^ [eV]	EA ^c^ [eV]	Eg ^c^ [eV]
(**1**)	1.14 (1.10) *	−1.46 (−1.44) *	−6.24	−3.64	2.60	2.58	−6.10	−3.20	2.90
(**2**)	0.79 (0.76) *	−0.68 (−0.67) *	−5.89	−4.42	1.47	1.69	−5.68	−4.10	1.58
(**3**)	0.67 (0.63) *	−0.93 (−0.88) *	−5.77	−4.17	1.60	1.68	−5.69	−4.09	1.60

^a^ Calculated from CV measurements (IP = −5.1 − Eox; EA = −5.1 − Ered; Eg (CV) = Eox (onset) − Ered (onset)); ^b^ obtained from UV–Vis using Eg = 1240/λ onset formula; ^c^ obtained from DFT; * obtained from DPV.

## Data Availability

The original contributions presented in this study are included in the article/Supplementary Material. Further inquiries can be directed to the corresponding author.

## Supplementary Materials

The following supporting information can be downloaded at https://www.mdpi.com/article/10.3390/molecules30132683/s1, Spectrum S1: ^1^H NMR spectrum of (**1**) (in CDCl_3_), Spectrum S2: ^13^C NMR spectrum of (**1**) (in CDCl_3_), Spectrum S3: ^1^H-^13^C HMQC NMR spectrum of (**1**) (in CDCl_3_), Spectrum S4: ^1^H-^13^C HMBC NMR spectrum of (**1**) (in CDCl_3_), Spectrum S5: ^1^H NMR spectrum of (**2**) (in CDCl_3_), Spectrum S6: ^13^C NMR spectrum of (**2**) (in CDCl_3_), Spectrum S7: ^1^H-^13^C NMR spectrum of (**2**) (in CDCl_3_), Spectrum S8: ^1^H-^13^C HMBC NMR spectrum of (**2**) (in CDCl_3_), Spectrum S9: ^1^H NMR spectrum of (**3**) (in CDCl_3_), Spectrum S10: ^13^C NMR spectrum of (**3**) (in CDCl_3_), Spectrum S11: ^1^H-^13^C HMQC NMR spectrum of (**3**) (in CDCl_3_), Spectrum S12: ^1^H-^13^C HMBC NMR spectrum of (**3**) (in CDCl_3_), Spectrum S13: ^1^H NMR spectrum of (**4**) (in CDCl_3_), Spectrum S14: ^13^C NMR spectrum of (**4**) (in CDCl_3_), Spectrum S15: ^1^H-^13^C HMQC NMR spectrum of (**4**) (in CDCl_3_), Spectrum S16: ^1^H-^13^C HMBC NMR spectrum of (**4**) (in CDCl_3_), Figure S1: Voltammograms for potentiostaticmethod (upper) and galvanostatic (down), Scheme S1: Schematic representation of the electrochemical synthesis reaction mechanism, CIP1 and CIP2 (cis intermediate product), TIP1 and TIP2 (trans intermediate product), Figure S2: Comparison of experimental (black line) and theoretical (black bar) absorption spectra, Figure S3: Theoretical absorption spectra, Table S1: Summary of photoluminescent properties of designed compounds dissolved in CH_2_Cl_2_ solution, Figure S4: TG curves for selected compounds:(**1**) for the substrates and (**2**) for *cis*-DBPBI, Figure S5: UV–Vis spectroelectrochemistry of (**1**) in DCM solution (c = 1 × 10^−5^ mol/L, as an inset on each graph, all potentials vs. Fc/Fc+ redox couple), Figure S6: UV–Vis spectroelectrochemistry of (**3**) in DCM solution (c = 1 × 10^−5^ mol/L, as an inset on each graph, all potentials vs. Fc/Fc+ redox couple).
