# Supplementary material for: Is the Greener Approach Better? Application of Electrochemistry in the Synthesis of Perylenediimides
Source: Molecules. 2025 Jun 21;30(13):2683. doi: 10.3390/molecules30132683 (PMC12251472; doi:10.3390/molecules30132683)
Supplement: Supplementary file 1 [file molecules-30-02683-s001.zip › molecules-3673583-supplementary.pdf]

Supporting Information

Patrycja Filipek[1]*, Agata Szlapa-Kula[1], Stanislaw Krompiec[1], Krzysztof Zemlak[2], Bartlomiej Kula[2], Karol Erfurt[3], Michal Filapek[1]*

[1]Institute of Chemistry, Faculty of Science and Technology, University of Silesia, Szkolna 9, 40-007 Katowice, Poland.
[2]Syntal Chemicals Sp. z o.o., ul Łabędzka 59. 44-121 Gliwice, Poland;
[3]Department of Chemical Organic Technology and Petrochemistry, Silesian University of Technology, B. Krzywoustego 4, 44-100 Gliwice, Poland; (K.E) karol.erfurt@polsl.pl

*Corresponding author at: Institute of Chemistry, University of Silesia, 9 Szkolna Str., 40-006 Katowice, Poland.
E-mail address:* patrycja.filipek@us.edu.pl;(P.Filipek) *michal.filapek@us.edu.pl;(M. Filapek)

Contents

1. NMR spectrum 3
2. Reaction voltamograms 10
3. DFT spectra 12
4. Photophysical properties 14

1. NMR spectrum

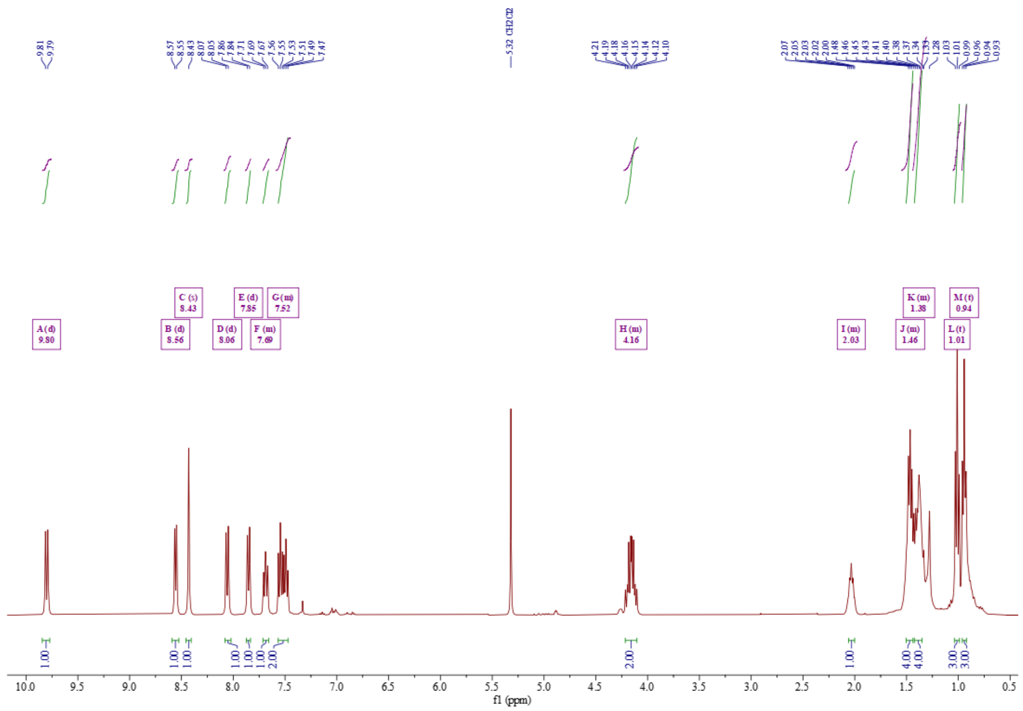

Spectrum S 1. ^{1}H NMR spectrum of **(1)** (in $CDCl_3$).

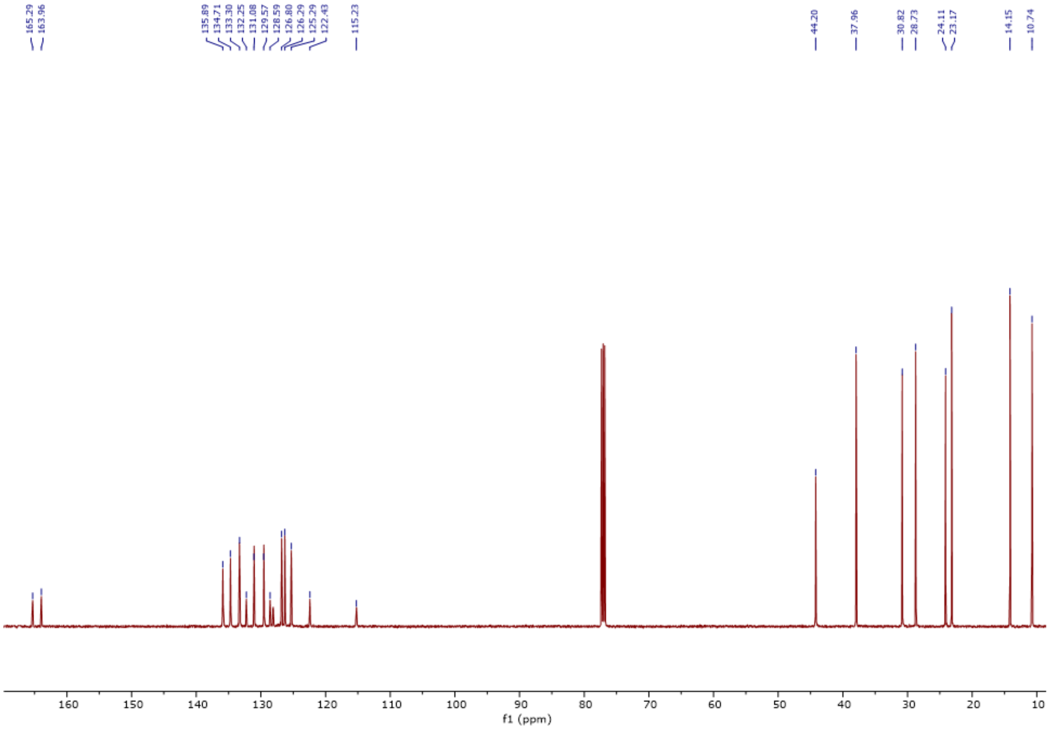

Spectrum S 2. ^{13}C NMR spectrum of **(1)** (in $CDCl_3$).

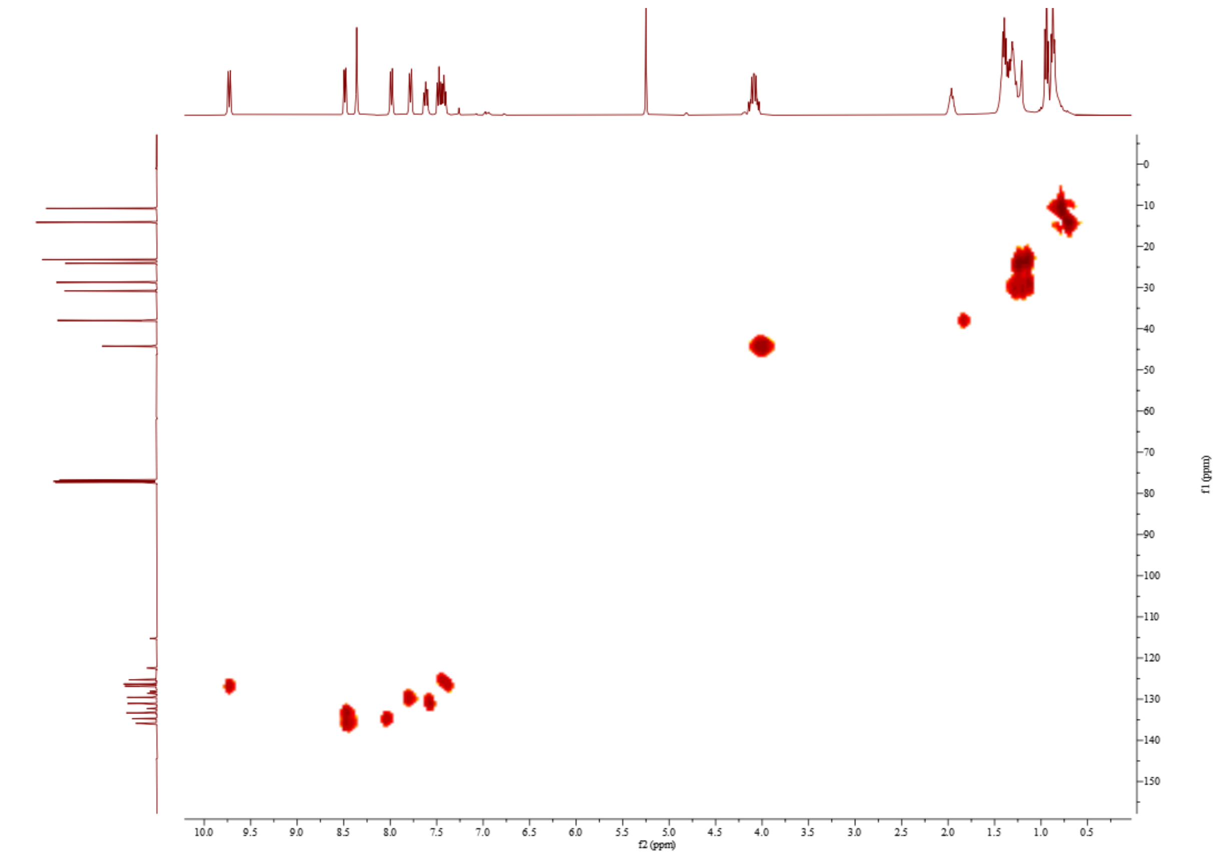

Spectrum S 3. ^{1}H-^{13}C HMQC NMR spectrum of **(1)** (in $CDCl_3$).

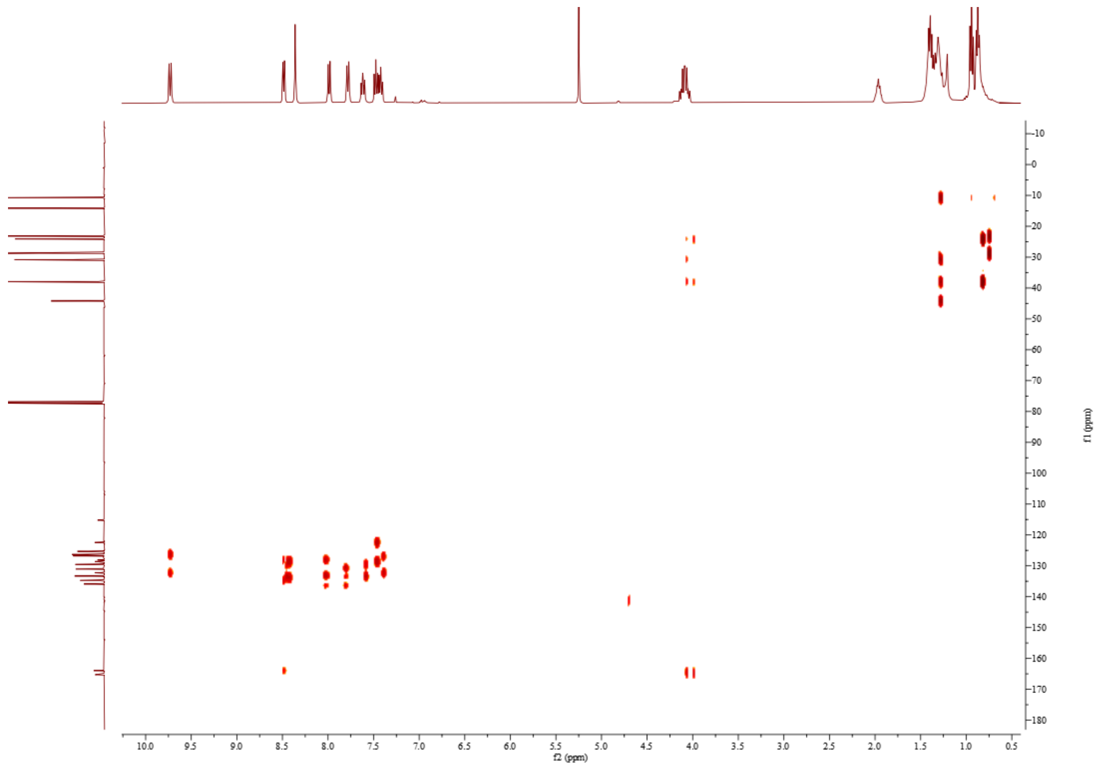

Spectrum S 4. ^{1}H-^{13}C HMBC NMR spectrum of **(1)** (in $CDCl_3$).

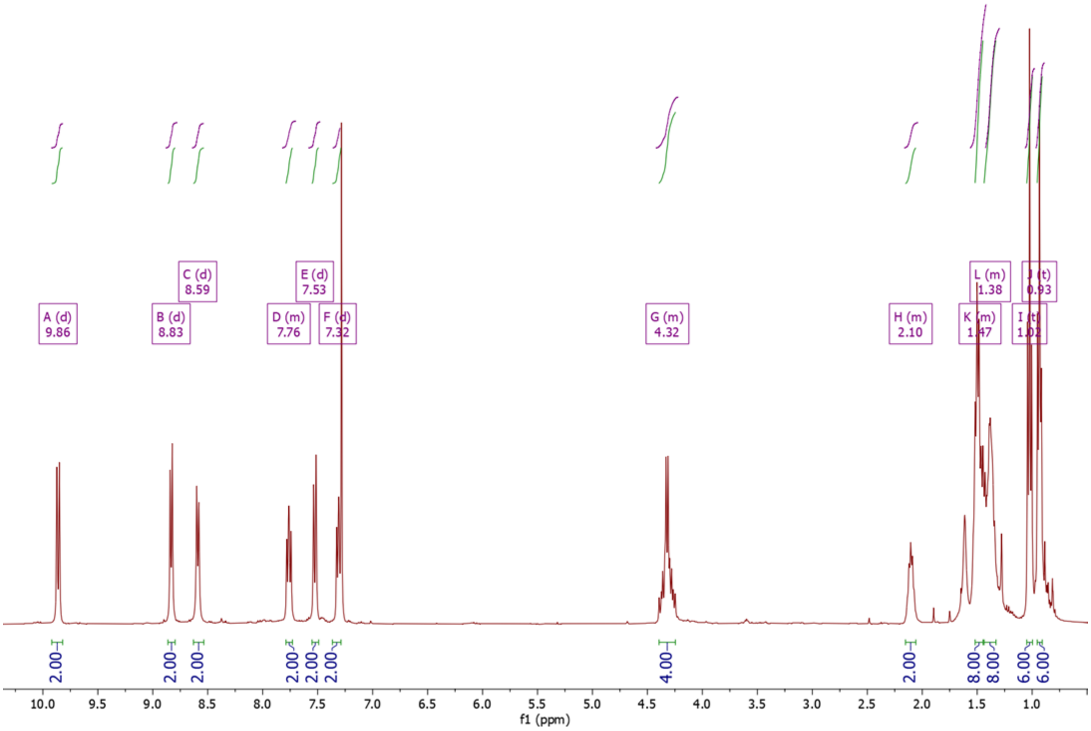

Spectrum S 5. ^{1}H NMR spectrum of **(2)** (in $CDCl_3$).

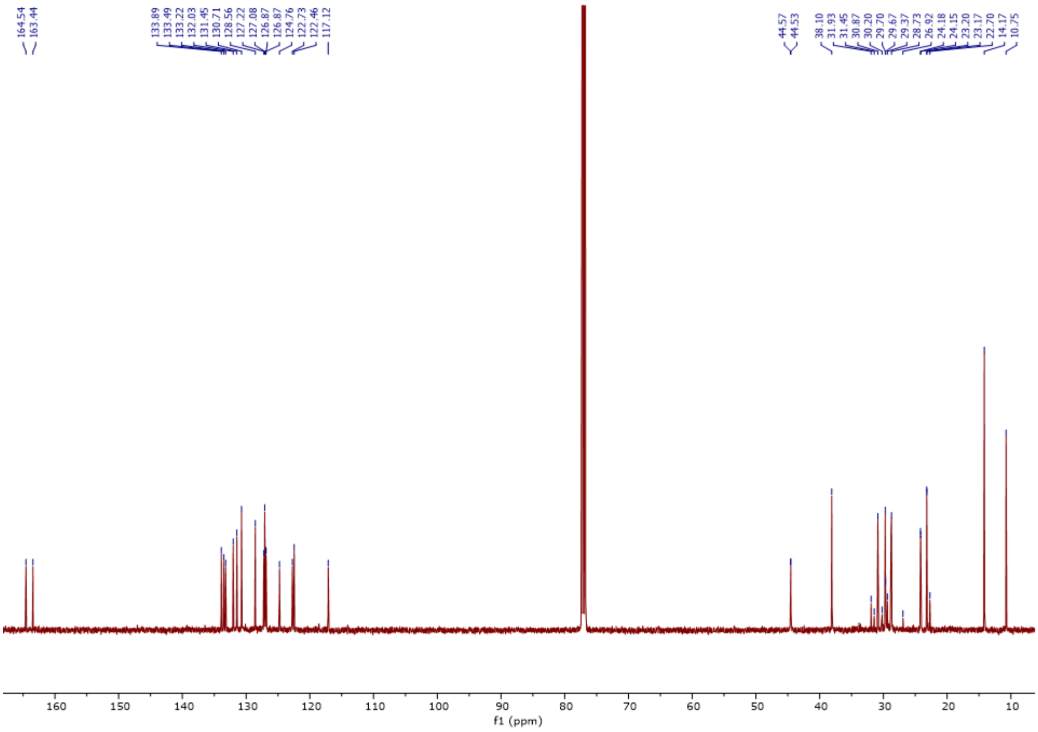

Spectrum S 6. ^{13}C NMR spectrum of **(2)** (in $CDCl_3$).

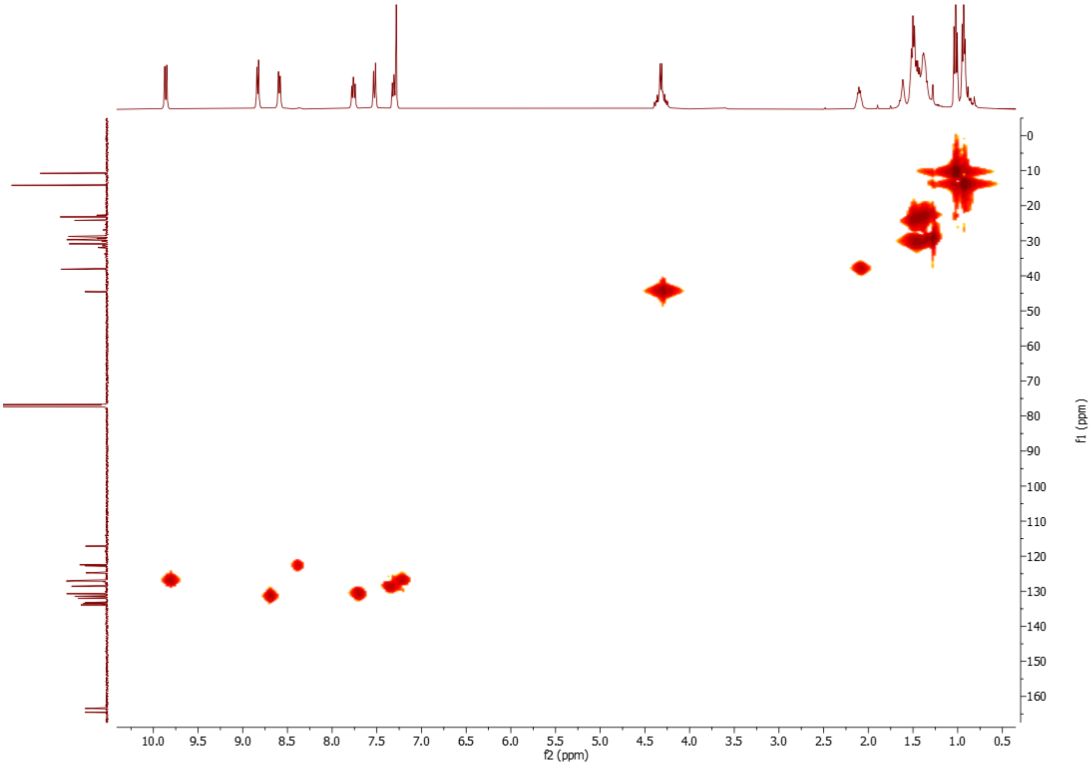

Spectrum S 7. ^{1}H-^{13}C NMR spectrum of **(2)** (in $CDCl_3$).

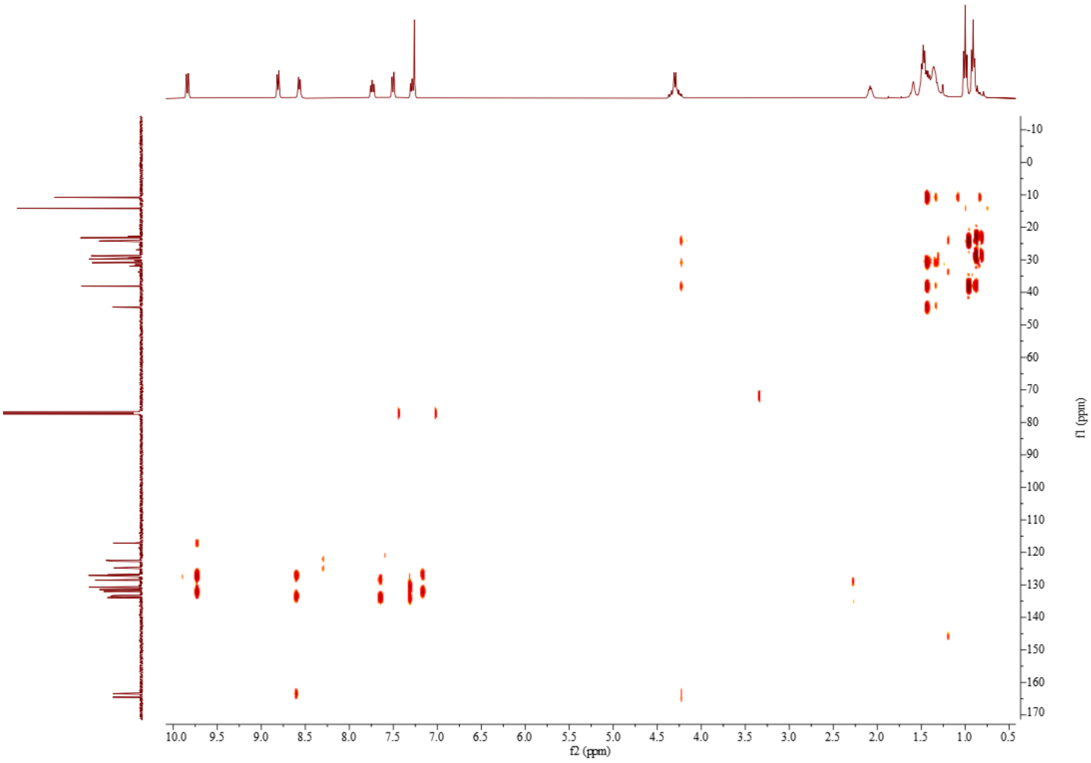

Spectrum S 8. ^{1}H-^{13}C HMBC NMR spectrum of **(2)** (in $CDCl_3$).

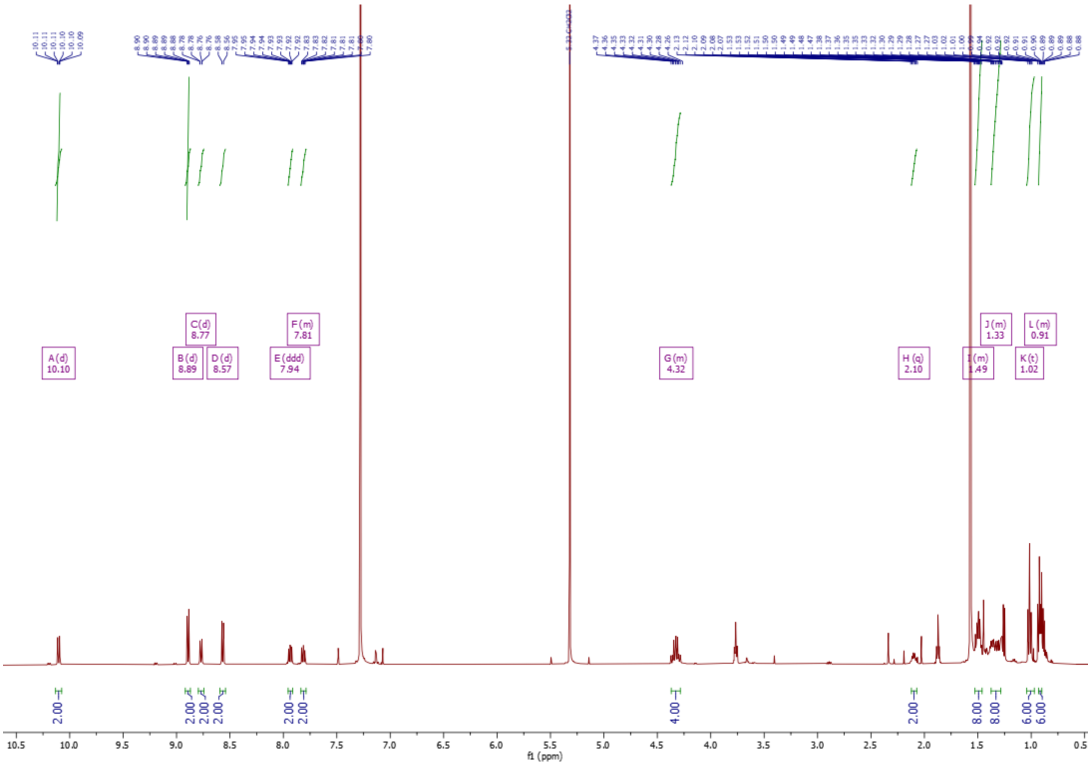

Spectrum S 9. ^{1}H NMR spectrum of **(3)** (in $CDCl_3$).

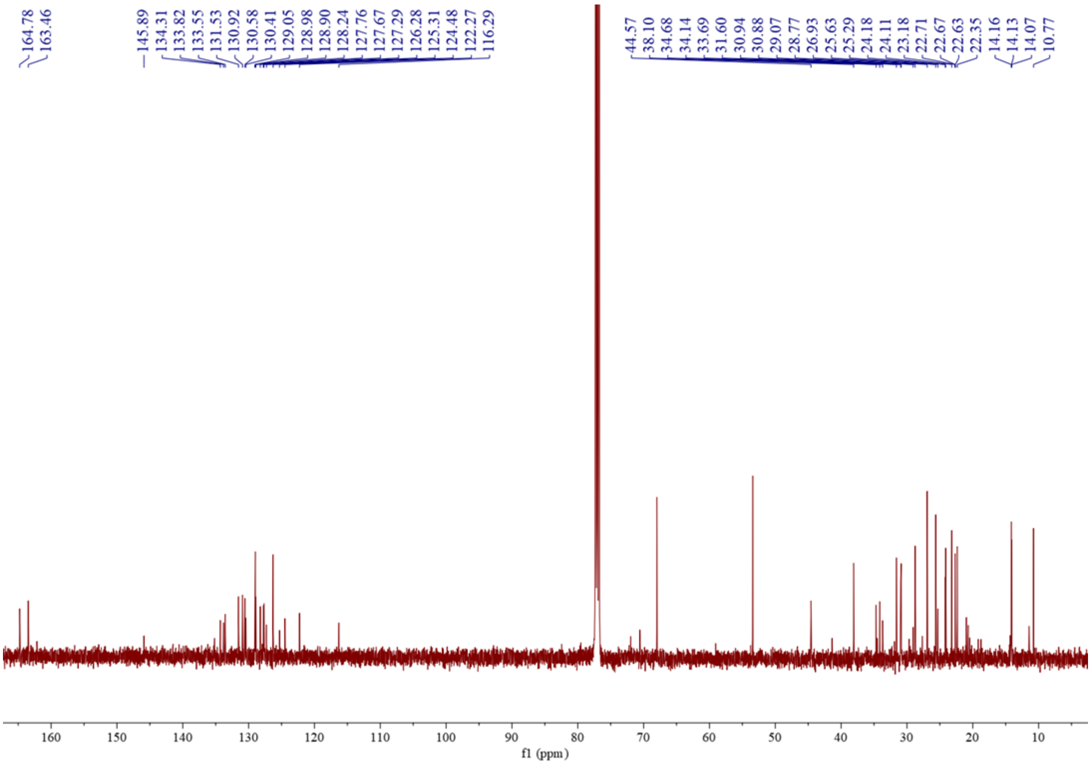

Spectrum S 10. ^{13}C NMR spectrum of **(3)** (in $CDCl_3$).

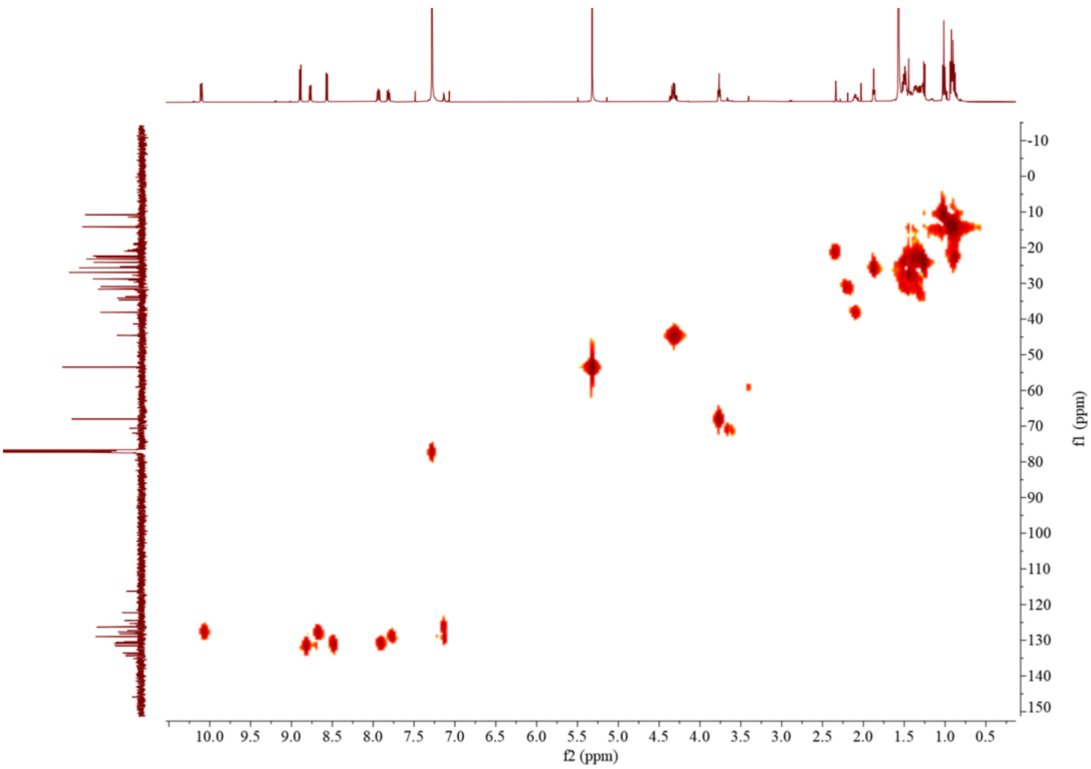

Spectrum S 11. ^{1}H-^{13}C HMQC NMR spectrum of **(3)** (in $CDCl_3$).

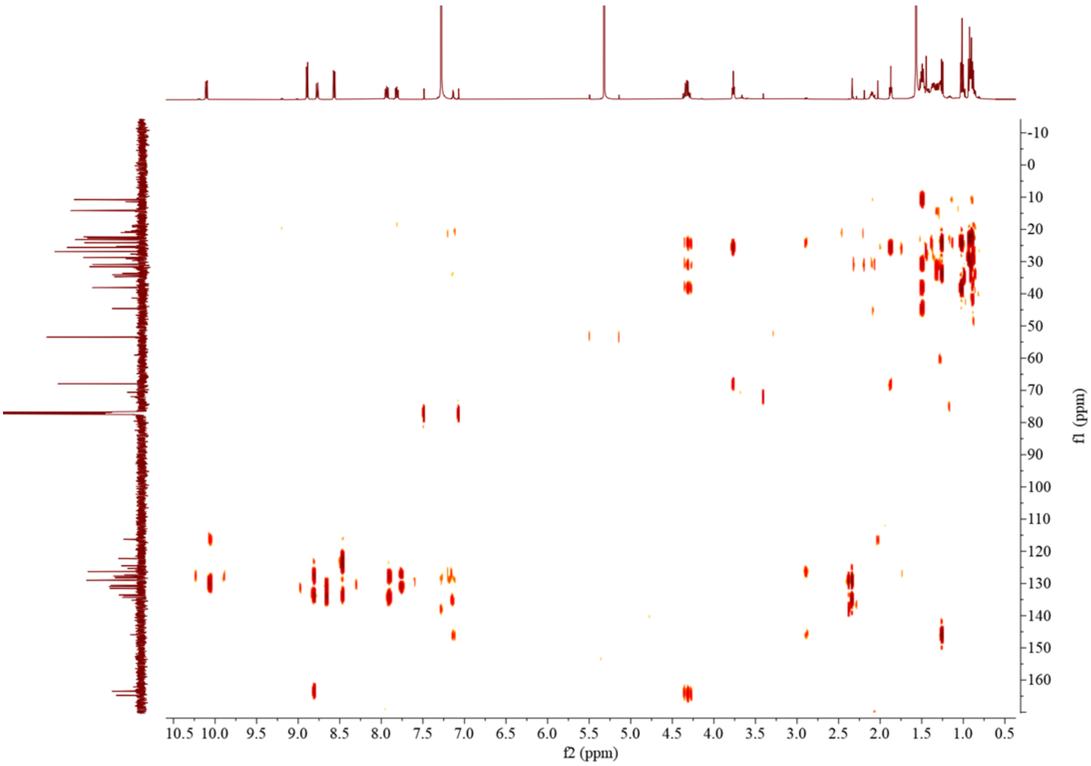

Spectrum S 12. ^{1}H-^{13}C HMBC NMR spectrum of **(3)** (in CDCl3).

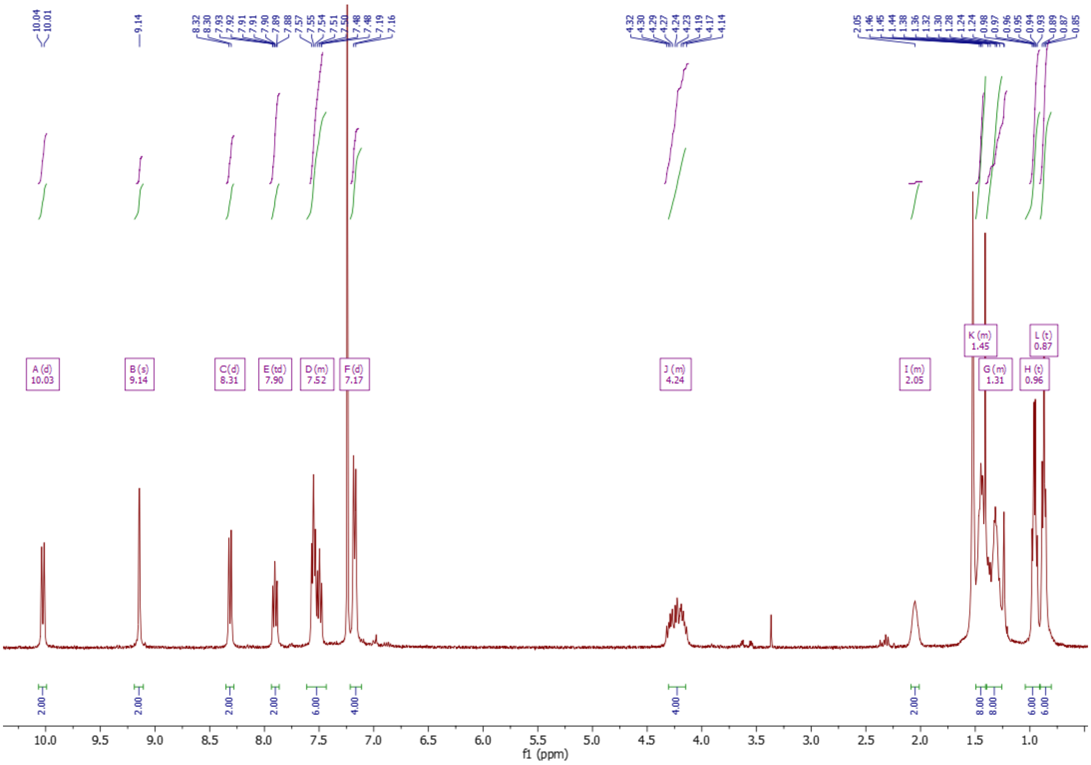

Spectrum S 13. ^{1}H NMR spectrum of **(4)** (in CDCl3).

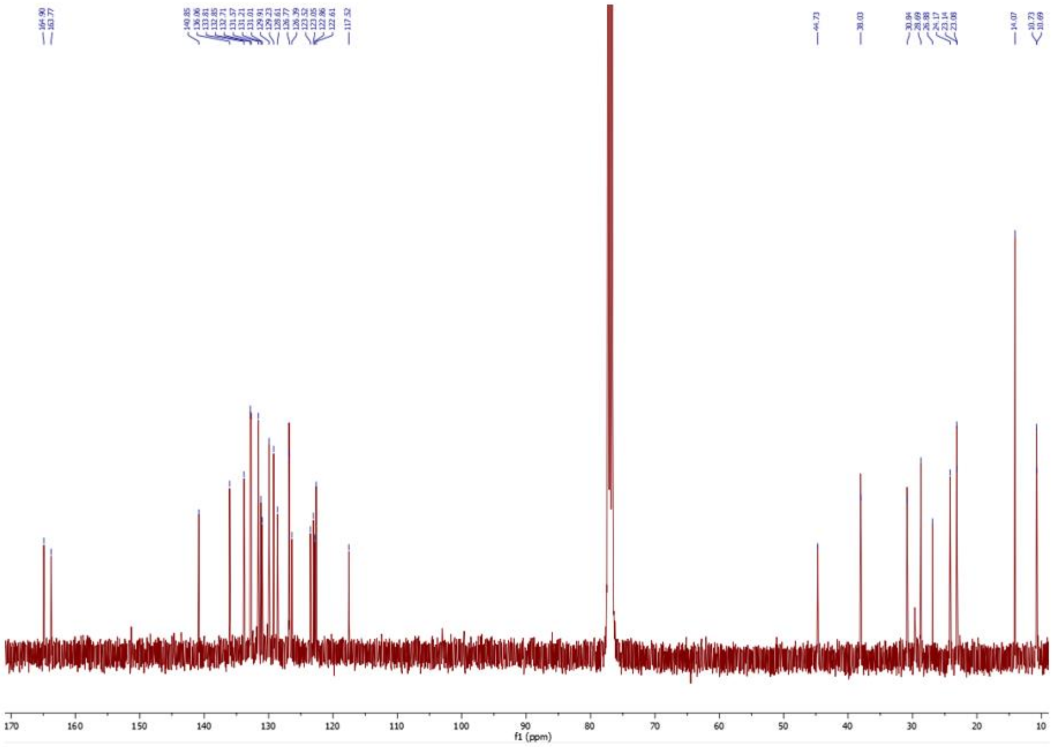

Spectrum S 14. ^{13}C NMR spectrum of **(4)** (in $CDCl_3$).

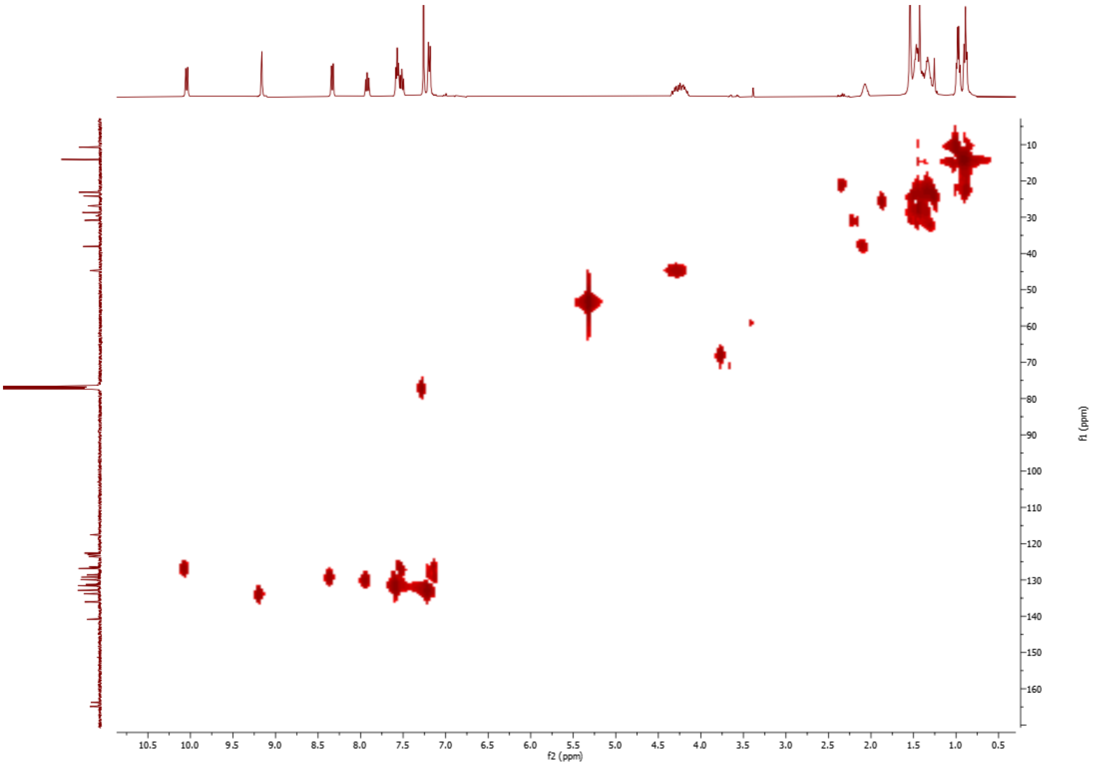

Spectrum S 15. ^{1}H-^{13}C HMQC NMR spectrum of **(4)** (in $CDCl_3$).

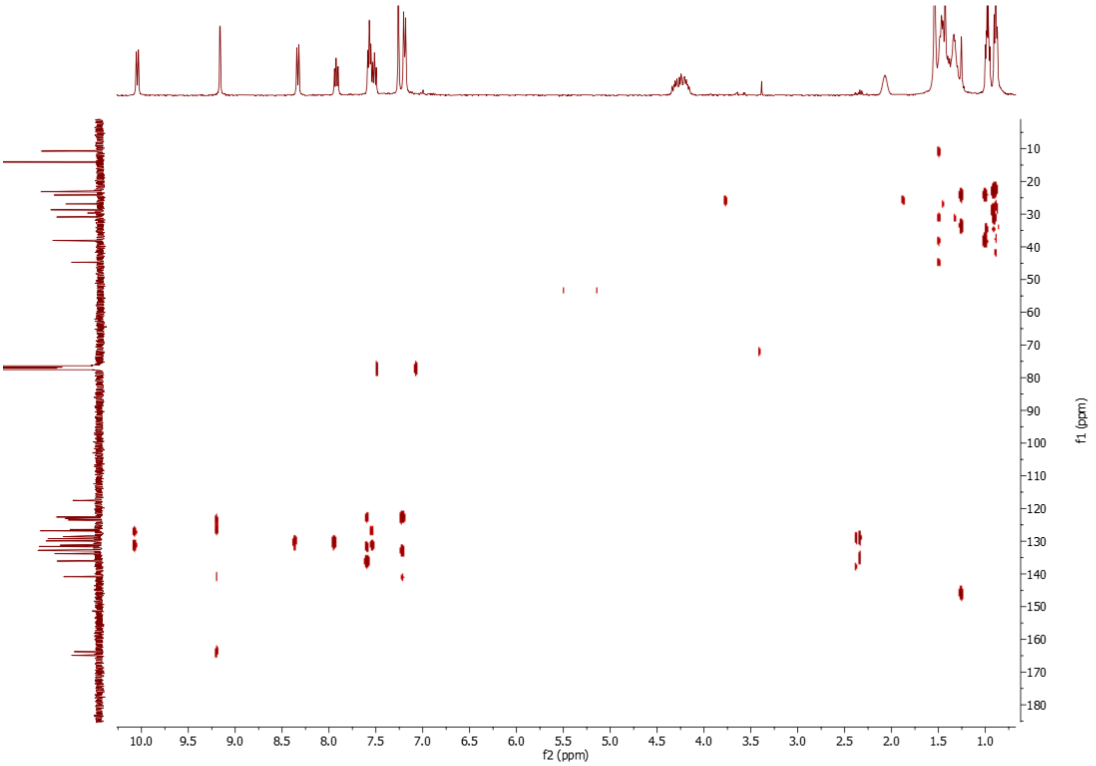

Spectrum S 16. ^{1}H-^{13}C HMBC NMR spectrum of **(4)** (in $CDCl_3$).

2. Reaction voltammograms

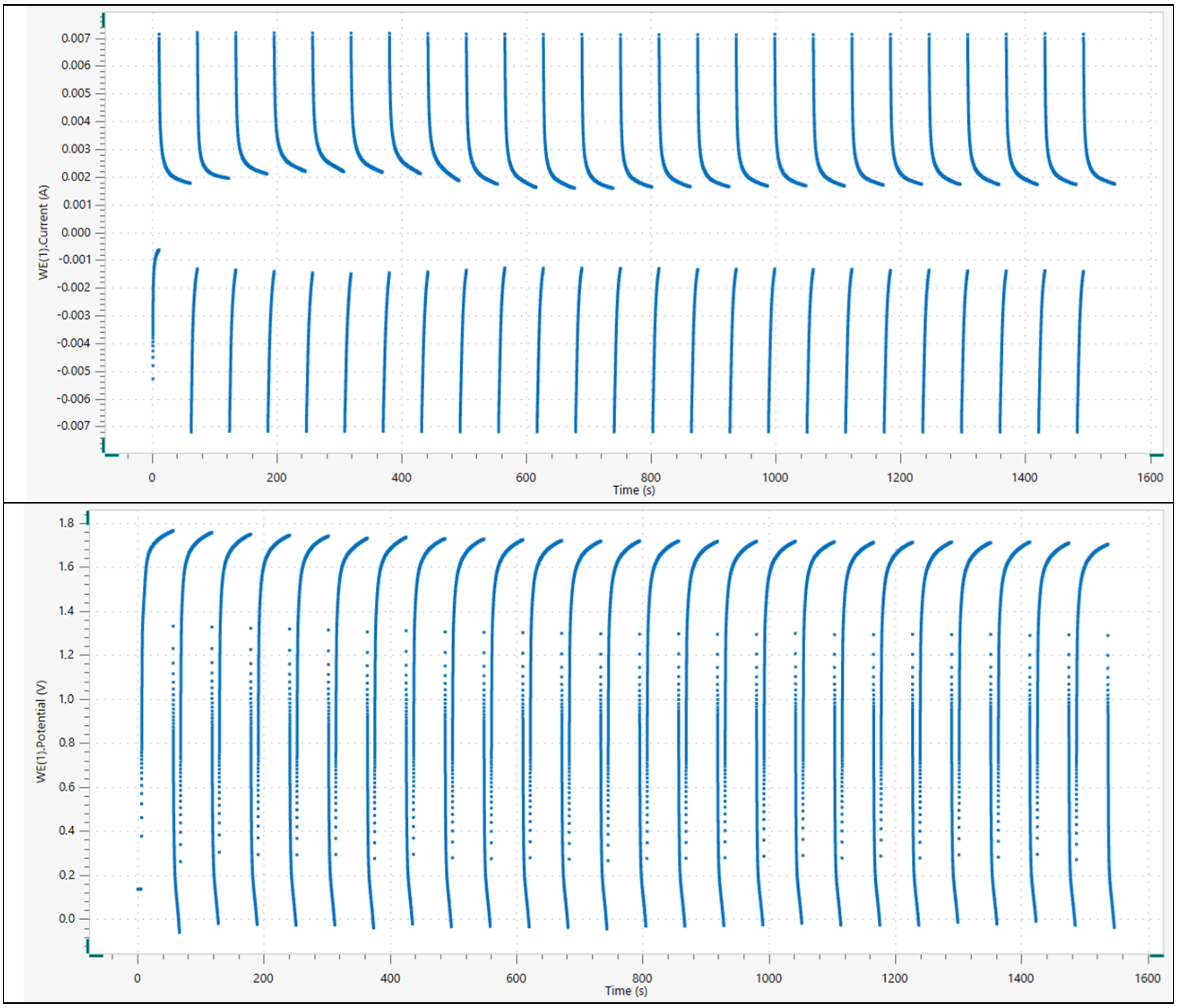

Figure S 1. Voltammograms for potentiostatic metohod (upper) and galvanotatic (down).

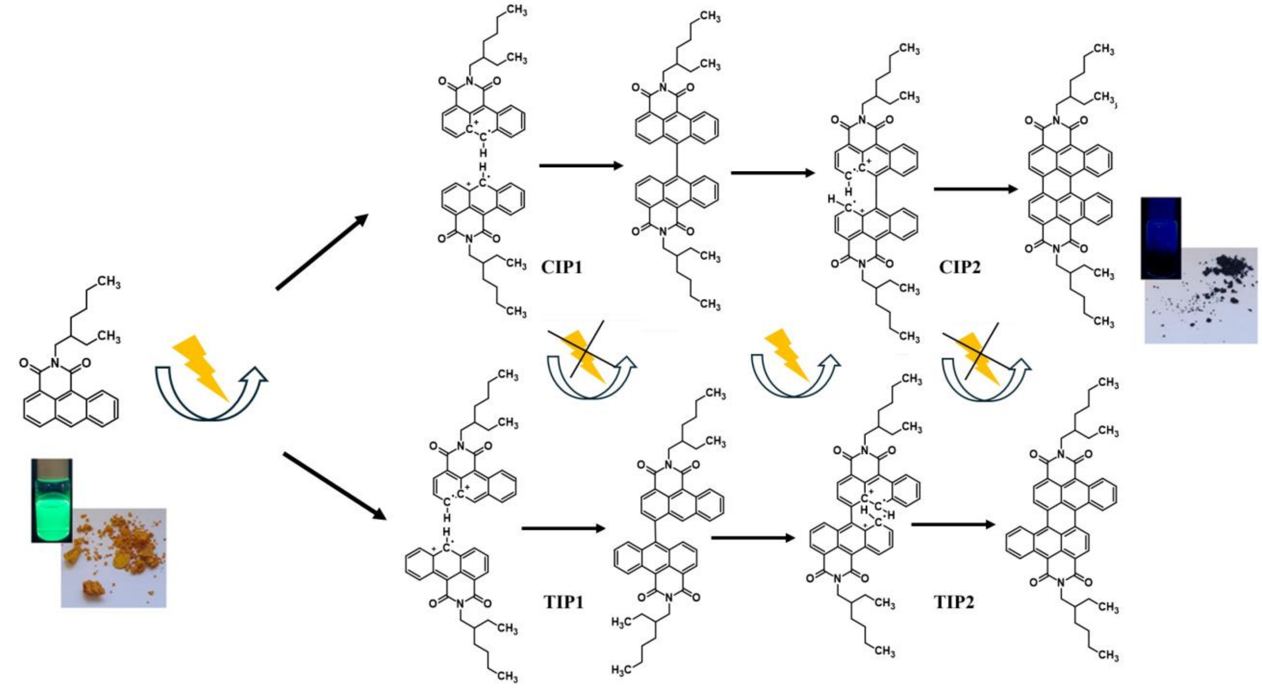

Scheme S 1. Schematic representation of the electrochemical synthesis reaction mechanism. CIP1 and CIP2 (cis intermediate product), TIP1 and TIP2 (trans intermediate product).

3. DFT spectra

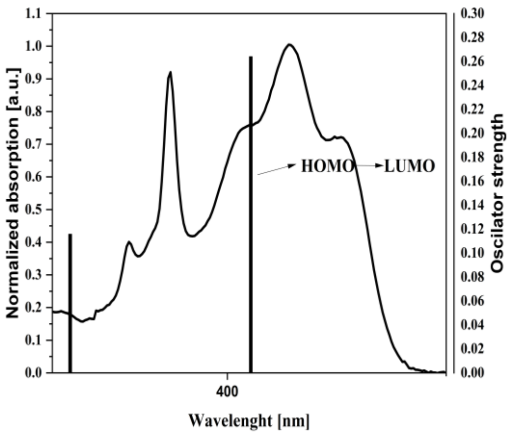

(1)

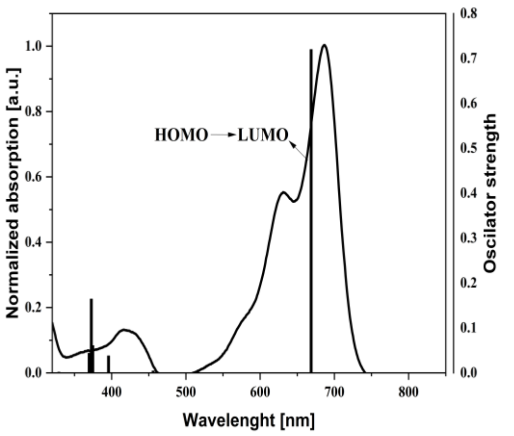

(2)

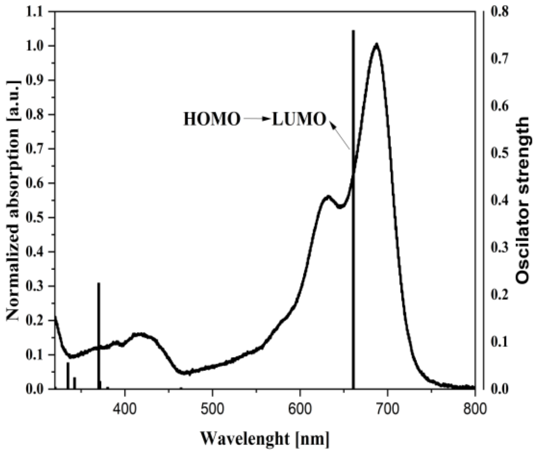

(3)

Figure S 2. Comparison of experimental (black line) and theoretical (black bar) absorption spectra.

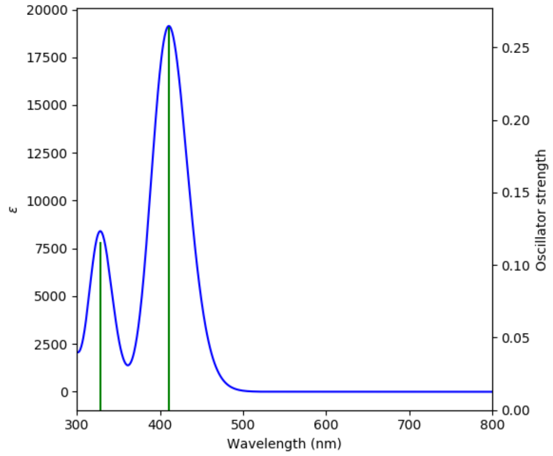

(1)

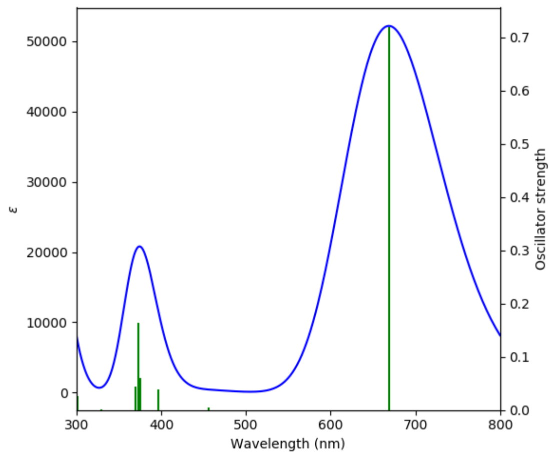

(2)

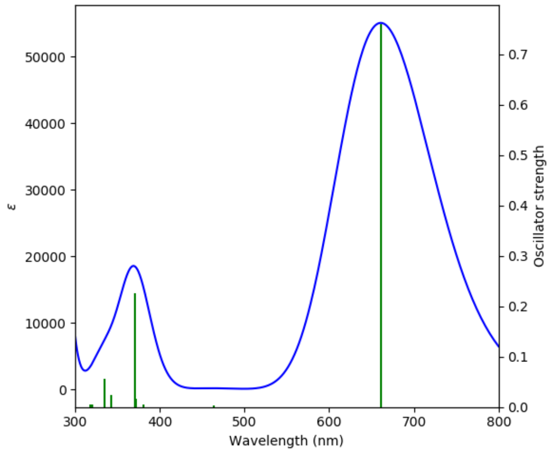

**(3)**

Figure S 3. Theoretical absorption spectra.

4. Photophysical properties

Table S 1. Summary of photoluminescent properties of designed compounds dissolved in CH_2Cl_2 solution

**Compound**	**λ [nm]**<br>**ε [M^{-1}×cm^{-1}]**	**Emission [nm]**	**Stokes shift [nm]**
**(1)**	354 (5.9×10^3)<br>374 (1.4×10^4)<br>428 (1.5×10^4)<br>452 (1.1×10^4)	486, 512	54
**(2)**	420 (3.9×10^3)<br>631 (1.7×10^4)<br>687 (3.1×10^4)	744	57
**(3)**	420 (1.5×10^3)<br>632 (5.4×10^3)<br>688 (9.6×10^3)	714	26

***** The underlined wavelengths were taken to register the emission spectrum

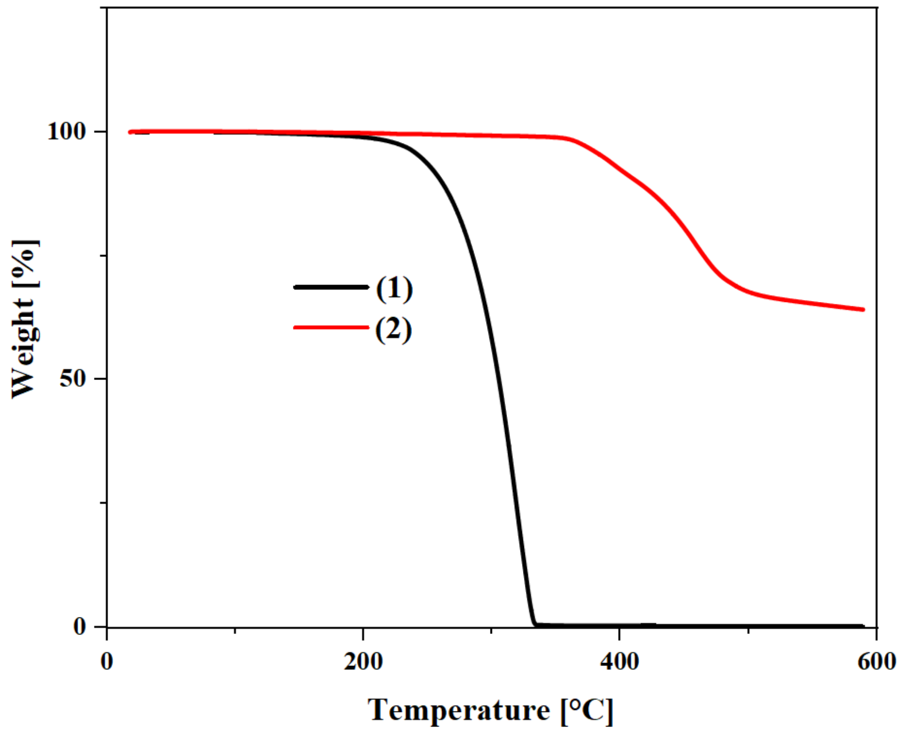

Figure S 4. TG curves for selected compounds. **(1)** for the substrates and **(2)** for cis-DBPBI.

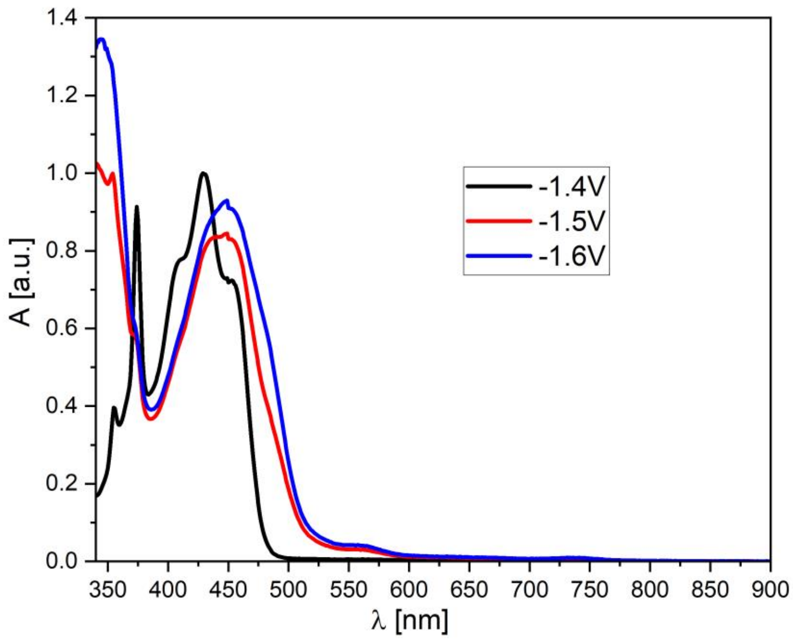

Figure S 5. UV-Vis spectroelectrochemistry of the **(1)** in DCM solution (c=1x10^{-5} mol/L, as an inset on each graph, all potentials vs Fc/Fc+ redox couple).

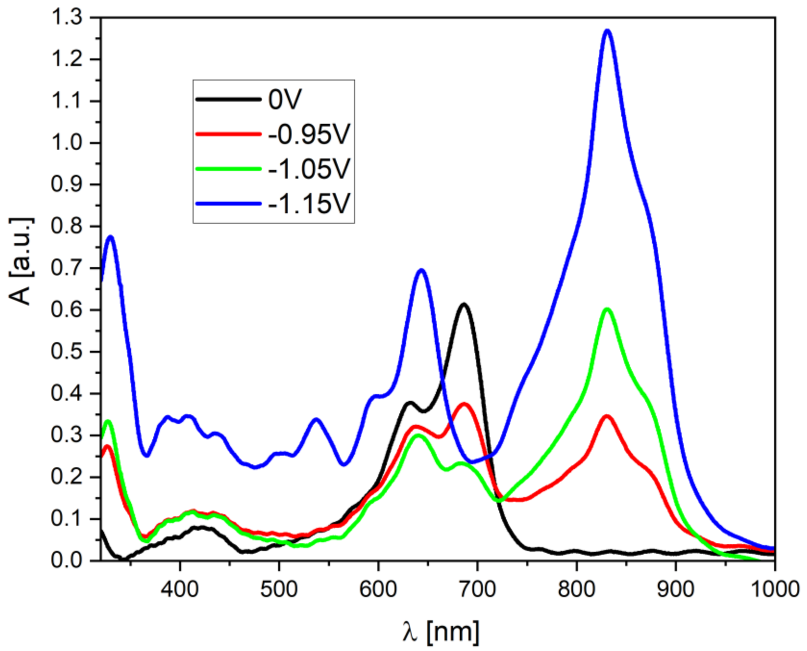

Figure S 6. UV-Vis spectroelectrochemistry of the **(3)** in DCM solution (c=1x10^{-5} mol/L, as an inset on each graph, all potentials vs Fc/Fc+ redox couple).
